# Physiological characterization and cold-responsive miRNA profiling of oil palm (*Elaeis guineensis* Jacq.) under cold stress

**DOI:** 10.3389/fpls.2026.1905015

**Published:** 2026-10-27

**Authors:** Zhihao Zhao, Qiufei Wu, Yajing Dou, Xiaohui Pan, Hongxing Cao, Xianhai Zeng, Lixia Zhou

**Affiliations:** 1 State Key Laboratory of Tropical Crop Breeding, Coconut Research Institute, Chinese Academy of Tropical Agricultural Sciences, Sanya/Wenchang, China; 2 Chinese Academy of Tropical Agricultural Sciences, Haikou, China; 3 Tangshan Key Laboratory of Numerical Computation and Simulation, School of Mathematics and Computational Sciences, Tangshan Normal University, Tangshan, Hebei, China

**Keywords:** cold stress, miRNA, oil palm, physiological response, small RNA sequencing

## Abstract

**Introduction:**

Cold stress seriously restricts the sustainable development of China’s oil palm industry. MicroRNAs (miRNAs), a class of small non-coding RNAs, act as key post-transcriptional regulators of gene expression and play essential roles in plant growth, development, and stress adaptation. Although they have been extensively characterized in various plant species under adverse environmental conditions, their regulatory functions in oil palm (*Elaeis guineensis* Jacq.) during cold stress responses remain largely unexplored.

**Methods:**

Seedlings of the thin shelled Tenera oil palm genotype were used in this study. Uniform aged seedlings were subjected to cold treatment at 8 °C, with sampling at 0 (26 °C), 0.5, 1, 2, 4, and 8 h, three biological replicates were included for each time point. We assessed physiological injury indicators including malondialdehyde, electrolyte leakage, and chlorophyll content. Eighteen independent small RNA libraries were sequenced on the Illumina HiSeq Xten platform, and cold responsive miRNAs and their predicted target genes were identified. Expression of 17 candidate miRNAs was further validated by qPCR.

**Results and discussion:**

Physiological measurements showed that with increasing cold stress, malondialdehyde accumulation and electrolyte leakage rate in leaves increased significantly, whereas chlorophyll content decreased continuously, indicating progressive impairment of cell membrane integrity. To this end, 18 independent small RNA sequencing libraries (three biological replicates for each of six experimental groups, including one normal temperature control (26 °C) and five cold-treatment time points (0.5, 1, 2, 4, and 8 h under 8 °C)) were constructed for small RNA profiling. A total of 191 unfiltered known miRNA candidates and 98 predicted novel miRNA candidates were initially identified by sequencing. Functional enrichment analysis revealed that the target genes of differentially expressed miRNAs were mainly enriched in transcriptional regulation pathways and sesquiterpenoid and triterpenoid biosynthesis. qPCR confirmed the expression dynamics of 17 cold responsive miRNAs, among which the negative regulation between the miR166 family and its predicted target genes *ATHB9-like* and *ATHB12*-*like* was observed based on expression correlation. Our data support the inference that miRNA dependent post transcriptional reprogramming participates in oil palm cold stress adaptation, rather than resolving the complete molecular mechanism. Further analysis indicated that temporal expression shifts of cold responsive miRNAs were consistent with dynamic changes in plant morphological, physiological, and biochemical indicators. Intersection analysis across the five cold treatment comparisons defined core cold responsive and stage specific sets for both conserved and novel miRNAs, and unbiased Mfuzz time series clustering classified all cold-responsive miRNAs into six temporal expression modules.

## 1 Introduction

Oil palm (*Elaeis guineensis* Jacq.) is a perennial tropical oil crop widely distributed between 10°S and 15°N latitude (Nugroho et al., 2024). Palm oil, produced from its fruit, is among the highest-yielding and most widely traded vegetable oils worldwide (Abubakar and Ishak, 2024). Oil palm thrives in warm and humid climates. In China, it is mainly cultivated in tropical areas such as Hainan and Yunnan provinces. Low winter temperatures can cause flower abortion and reduce yield, thereby severely affecting palm oil production (Imran et al., 2026). Therefore, low temperature is a key factor limiting the growth and development of oil palm.

In recent years, increasing attention has been paid to the cold tolerance of oil palm. Multiple studies have shown that oil palm adapts to cold stress through morphological changes, osmotic regulation, activation of antioxidant enzyme systems, and hormone signaling pathway regulation (Li et al., 2019; Zhou et al., 2022, 2025; Imran et al., 2026). At the molecular level, SSR marker analysis has been used to identify genes associated with cold response in oil palm (Xiao et al., 2014), and transcriptome analysis has revealed the gene expression regulatory network under cold conditions, leading to the identification of numerous genes involved in cold response (Lee et al., 2024; Saand et al., 2022). Despite these advances, the regulatory mechanisms underlying cold response in oil palm remain to be further explored. In addition to the aforementioned physiological changes, microRNAs (miRNAs) play important roles in plant responses to biotic and abiotic stresses and have become a research hotspot in recent years (Singh et al., 2023; Islam et al., 2022; Chen et al., 2025). Studies have shown that miRNAs participate in regulating plant cold tolerance by affecting the cleavage of cold responsive genes or inhibiting their translation. For example, miRNAs have been confirmed to be involved in cold stress responses in rice (Shen et al., 2025; Gao et al., 2025), poplar (Yang et al., 2026), and other plants (Zhu et al., 2023; Abla et al., 2019). Many miRNA target genes encode transcription factors, suggesting that miRNAs may occupy key positions in the regulatory network of cold stress (Yang et al., 2026; Chen et al., 2022). Therefore, identifying miRNAs and their target genes is helpful for revealing the molecular mechanisms of miRNA-mediated cold stress responses.

Currently, some conserved miRNAs and their target genes have been identified in oil palm (Zhou and Yarra, 2023; Wu et al., 2025). Certain miRNAs have been confirmed to play important regulatory roles in salt stress response and floral organ development in oil palm (Salgado et al., 2021, 2022). However, systematic studies on miRNAs under cold stress in oil palm remain limited. Compared with other crops, research on miRNA expression profiles in oil palm under cold stress is still relatively weak. In this study, we used high-throughput sequencing technology to analyze the expression profiles of miRNAs in oil palm under different cold stress conditions, predicted the target genes of cold-responsive miRNAs, and compared the physiological indicators (malondialdehyde content, electrical conductivity, and chlorophyll content) between cold stressed and control plants. We aim to characterize miRNA expression shifts under cold exposure at the transcript level, rather than fully dissect the complete cold response regulatory mechanism. Our outputs will deliver priority miRNA candidates (including Eg-miR166, Eg-miR393, Eg-miR156, Eg-miR444) for future functional assays and molecular breeding for cold tolerance in oil palm and provide foundational reference data for understanding miRNA associated cold stress responses in this tropical crop.

## 2 Materials and methods

### 2.1 Plant materials and cold stress treatment

Seedlings of the Tenera fruit form (F_1_ hybrid derived from Dura × Pisifera crosses) of African oil palm (*Elaeis guineensis* Jacq.) were obtained from the Germplasm Nursery of the Coconut Research Institute, Chinese Academy of Tropical Agricultural Sciences (Wenchang, Hainan, China (19.63°N, 110.75°E)). Tenera is the dominant cultivated fruit form for commercial palm-oil production in China and tropical Southeast Asia, it is not a botanical variety but an F_1_ hybrid fruit type derived from Dura × Pisifera crossing. Seed derived Tenera seedlings retain residual genetic heterogeneity even from a single seed lot, because this hybrid fruit form is maintained via seed propagation rather than vegetative cloning. In this study, ten-month-old uniform-sized seedlings with eight fully expanded functional leaves were selected. Each seedling was grown in a 22 cm-diameter plastic pot filled with a substrate of peat soil: coconut coir: perlite (2:2:1, v/v/v). Seedlings were watered every two days to maintain soil field capacity, and compound liquid fertilizer was applied biweekly.

Before cold stress imposition, seedlings were acclimated in controlled-growth chambers for 14 days under 26 °C, a 16 h light/8 h dark photoperiod, a PPFD of 320 μmol m^-2^ s^-1^, and 65 ± 5% relative humidity. Cold stress was imposed by directly transferring intact potted seedlings into a separate pre-cooled growth chamber set to 8 °C, the identical 16 h light/8 h dark photoperiod, PPFD (320 μmol m^-2^ s^-1^), and relative humidity (65 ± 5%) were maintained throughout the cold stress period. Cold stress treatment was initiated at 08:00 (local clock time). Harvest sampling was performed at 08:00 (CK, 0 h baseline control), 08:30 (Cold 0.5 h), 09:00 (Cold 1 h), 10:00 (Cold 2 h), 12:00 (Cold 4 h), and 16:00 (Cold 8 h). For each plant, the spear leaf (youngest fully expanded spear shaped leaf) was harvested as the experimental tissue.

All experimental seedlings were selected from a single seed lot of Tenera to minimize genetic variation, but residual genetic differences among seed siblings cannot be fully eliminated. Importantly, destructive single-time-point sampling was applied: each seedling was harvested only at one designated time point. Therefore, intrinsic genetic variance among individual seedlings is partially confounded with cold treatment effects, clonal vegetatively propagated material would eliminate this source of noise and would be preferable for future repeated-measures sampling designs. For cold stress treatment, a total of 18 seedlings were used for destructive sampling. Three seedlings grown continuously at 26 °C served as the normal temperature control (CK). For cold stress treatments, three independent seedlings were assigned exclusively to each time point (0.5, 1, 2, 4, and 8 h at 8 °C, three biological replicates per time point), giving 15 cold treated seedlings in total. Each seedling was harvested only once at its designated sampling time, no plant was sampled repeatedly across multiple time points. Spear leaves were harvested from each seedling at the corresponding time point, immediately snap-frozen in liquid nitrogen, and stored for subsequent analysis. To evaluate sample reproducibility across the 18 independent small RNA sequencing libraries, we performed Pearson correlation analysis and principal component analysis (PCA). The correlation heatmap and PCA plot for all 18 libraries are provided in **Supplementary Figure S1** and **Supplementary Figure S2**, respectively.

No plant material, leaf tissue, RNA samples or small RNA sequencing datasets in the present study overlap with Imran et al. (2026). The oil palm germplasm used in this work is distinct from that reported in Imran et al. (2026). While transcriptome reference reads were retrieved from Wu et al. (2025), all seedlings, tissue sampling, RNA extraction and 18 small RNA sequencing libraries were newly generated exclusively for this miRNA profiling study.

### 2.2 Determination of electrical conductivity, chlorophyll and malondialdehyde concentrations

To assess cold stress tolerance, the physiological and biochemical properties of leaf tissues from all experimental groups were evaluated. All treatments were performed in triplicate. MDA concentration was quantified using specific Microassay Kits (Solarbio, Beijing, China). Leaf chlorophyll concentration was determined as previously described (Amsellem and McKey, 2006). Leaf conductivity was measured according to Luo et al (Luo and Huang, 2012).

Leaf color parameters were additionally determined on intact fresh spear leaves at the same sampling time-points used for physiological assays, referring to the colorimetric methodology for plant stress monitoring described by Bacci et al. (1998). Briefly, CIELAB color coordinates (*L*, *a*, *b*) were measured with a Minolta CR-200 portable colorimeter. The instrument was set to illuminant C and d/0° viewing geometry.

Three basic CIELAB coordinates are defined as follows. *L* represents lightness, ranging from 0 (black) to 100 (white). *a* indicates the red-green axis, positive values indicate redness, and *b* denotes the yellow-blue axis, positive values indicate yellowness, and negative values indicate blueness. Total color difference (*ΔE*_ab_), was calculated to evaluate the overall color difference between cold-stressed leaves and non-stressed control leaves. The calculation formula for total color difference is:


$$\Delta E*\text{ab}=\sqrt{{\left(\Delta L*\right)}^{2}+{\left(\Delta a*\right)}^{2}+{\left(\Delta b*\right)}^{2}}$$

In this formula, *ΔL* = *L*_treatment_ − *L*_CK_, representing the lightness difference between each cold-stress treatment group and the normal-temperature control (CK).

*Δa* = *a*_treatment_ − *a*_CK_, representing the red-green coordinate difference between each cold-stress treatment group and CK.

*Δb* = *b*_treatment_ − *b*_CK_, representing the yellow-blue coordinate difference between each cold-stress treatment group and CK.

Hue angle (h°) and chroma (C) were further calculated to reflect leaf tone and color saturation.

The hue angle h°(0-360°) was computed using the four-quadrant corrected arctan (b*, a*) function, rather than simple arctangent arctan (b*, a*), to account for negative a* value in green leaf tissue.


$${\text{ho}}^{=}\text{atan}2\left(b*,a*\right)\times \frac{180}{\pi }$$

Values were converted from radians to degrees (°). For green plant tissues, a* is typically negative, and valid hue angles for green leaves fall within 120°–180°.

The formula for chroma:


$${\text{C∗}}^{=}\sqrt{{\text{a}}^{2}+{\text{b}}^{2}}.$$

which indicates the saturation or vividness of leaf color.

Three biological replicates were included for each treatment. Multiple technical measurements were performed on the surface of each leaf. For L, a*, b*, h°, and chroma C, data from all six experimental groups (CK and five cold stress time points) were subjected to one way ANOVA followed by Tukey’s *post-hoc* test. For *ΔE*_ab_ (total color difference relative to CK), the CK value is mathematically constrained to zero by definition and was excluded from ANOVA, only the five cold stress treatment groups were statistically compared.

### 2.3 RNA extraction and transcriptomic analysis

Total RNA was extracted from oil palm leaf tissue using the TRIzol reagent kit (Invitrogen, Carlsbad, CA, USA) following the manufacturer’s protocol. RNA quality was evaluated using 1.2% agarose gel electrophoresis, and RNA molecules ranging from 18 to 30 nt were enriched by 15% denaturing polyacrylamide gel electrophoresis (PAGE, 8M urea, 1 × TBE buffer). Gel fragments corresponding to 18–30 nt RNA were excised, crushed, and eluted in 0.3 M NaCl solution at 4 °C overnight, RNA was subsequently precipitated with ethanol and resuspended in RNase-free water for downstream library preparation. Custom adapter sequences used for library construction: 5′-adapter: 5′-GUUCAGAGUUCUACAGUCCGACGAUC-3′ 3′-adapter: 5′-rApp-TGGAATTCTCGGGTGCCAAGG-3′-C3-spacer. Subsequently, 3’ adapters were ligated to size selected small RNAs, and RNAs of 36–48 nt (small RNA + 3′-adapter) were enriched via a second round of 15% denaturing PAGE gel purification. The 5’ adapters were ligated to the recovered RNAs. The ligation products were reverse transcribed, followed by 12 cycles of PCR amplification (98 °C 10 s; 60 °C 30 s; 72 °C 15 s; final extension 72 °C 5 min). PCR products of 140–160 bp were enriched by 8% native PAGE gel, excised, eluted, precipitated, and resuspended to generate the final cDNA library, which was sequenced using Illumina HiSeq Xten platform by Gene Denovo Biotechnology Co. (Guangzhou, China).

For library construction, RNA from each individual biological replicate was processed separately. No sample pooling was conducted, 18 independent small RNA cDNA libraries were generated for subsequent Illumina sequencing. Small RNA sequencing data were processed using the following software: Cut adapt (version 4.1) for adapter trimming with parameters -a TGGAATTCTCGGGTGCCAAGG -m 18 -M 30 --quality-cutoff 20, and FASTX-Toolkit (version 0.0.14) for quality filtering with parameters -q 20 -p 90.

For sample reproducibility assessment, normalized miRNA read counts (TPM, transcripts per million) from 18 small RNA libraries were used as input for downstream multivariate analysis. Pearson pairwise correlation coefficients between all biological libraries were computed and visualized as correlation heatmap. Principal component analysis (PCA) was performed on the same TPM normalized miRNA expression matrix to explore global miRNA expression variance across control and cold stress time points. Both Pearson correlation and PCA were implemented using R software (v4.2.1), pheatmap package was used for heatmap visualization, and prcomp function was applied for PCA computation.

### 2.4 miRNA annotation and target gene prediction

Clean tags were first aligned against the GenBank (Release 209.0) and Rfam (Release 11.0) databases to remove rRNA, scRNA, snoRNA, snRNA, and tRNA fragments. The remaining sequences were then aligned to the oil palm reference genome (EG11, https://www.ncbi.nlm.nih.gov/datasets/genome/GCF_000442705.2/), and sequences mapping to exons, introns, or repeat regions were discarded to eliminate potential mRNA-derived degradation fragments. For known miRNA annotation, only plant-derived mature miRNA sequences from miRBase Release 22 were used, animal, fungal and viral miRNA entries were excluded to avoid cross-kingdom mis-annotation. Known oil palm miRNAs were identified using this plant-only miRBase subset. Sequences without hits in miRBase Release 22 were further aligned against miRNA sequences from closely related palm species to annotate conserved plant miRNAs.

For miRNA sequence alignment, Bowtie (version 1.2.3) was used with parameter -v 0 (zero mismatches). Multiple valid alignments were retained for subsequent filtering. Following Axtell and Meyers (2018) plant-miRNA criteria, sequences with length >23 nt were excluded from known-miRNA calls to filter pervasive 24-nt heterochromatic siRNA contamination. Clean tags were aligned against the plant-only subset of miRBase (Release 22), Rfam (Release 11.0), and GenBank (Release 209.0).

For novel miRNA prediction, clean small-RNA reads were mapped to the oil palm reference genome (EG11, https://www.ncbi.nlm.nih.gov/datasets/genome/GCF_000442705.2/). miRDeep-P2 (version 2.0.2), a plant-optimized miRNA prediction tool, was used as the primary predictor. Novel miRNA candidates were strictly filtered following the standardized plant-miRNA annotation criteria described by Axtell and Meyers (2018): (1) the miRNA/miRNA duplex possesses a characteristic 2-nucleotide 3′ overhang, (2) mature miRNA reads predominantly map to one arm of the predicted hairpin precursor, (3) the hairpin precursor exhibits stable secondary structure with adequate minimum free energy (MFE), (4) spurious signals originating from mRNA degradation or siRNA loci are excluded. Auxiliary evidence was collected using miRDeep2 (version 2.0.1.2, non-default parameters: -d 50 -m 10 -M 5000 -p 500000 -r 20 -t Palm), Mireap (version 0.2, parameters: -l 18 -L 26 -d 200 -D 350 -m 15 -M 30 -s 2 -e -25), and miRdentify (version 1.2, parameters: -m 18 -M 26 -s 10 -t 0.5 -c 2). Candidates passing all Axtell and Meyers (2018) criteria, with miRDeep-P2 score ≥ 10 and MFE ≤ −25 kcal/mol, and supported by at least one auxiliary prediction tool, were retained as high-confidence novel miRNAs.

For target gene prediction, the following criteria and software were employed. psRNATarget (version 2020.1) was used with parameters: score threshold = 3.0, expectation value ≤ 3.5, target accessibility maximum = 25. Additionally, TargetFinder (version 1.6) was applied with parameters: -c 4 -m 5 -s 0.5 -n 2, and miRanda (version 3.3a) was used with parameters: -sc 150 -en -20 -strict. Only target genes predicted by at least two of the three programs and satisfying the mismatch criteria described below were retained. The specific mismatch criteria for target prediction were: (1) a maximum of four mismatches between the small RNA and its target, with G-U base pairs weighted as 0.5 mismatches, (2) no more than two consecutive mismatches within the miRNA/target duplex, (3) no adjacent mismatches permitted at positions 2–12 from the 5’ end of the miRNA, (4) no mismatches allowed at positions 10–11, (5) a maximum of 2.5 weighted mismatches within the 5’ seed-like region (positions 1–12), and (6) the minimum free energy (MFE) of the miRNA/target duplex must be at least 75% of the MFE of the miRNA bound to its perfectly complementary sequence, calculated using RNAduplex (ViennaRNA package version 2.4.18) with parameters -d 2 -noLP.

For transcriptome read alignment used as a reference, HISAT2 (version 2.2.1) was employed with parameters: --dta -k 5 --pen-noncansplice 10 --mp 1,0 --sp 1,0 --min-intronlen 20 --max-intronlen 50,000. These transcriptome reads were generated in house from oil palm leaf samples in the present study and deposited in the China National GeneBank Database (CNGB) under accession number CNP0006920. The resulting SAM files were processed with samtools (version 1.15.1) for sorting and indexing.

KEGG enrichment analysis was performed, *p*-values were adjusted for multiple testing using Benjamini-Hochberg FDR correction to generate q-values. Rich-Factor was calculated as (number of target genes in pathway)/(total background gene number annotated to this KEGG pathway).

Gene Ontology (GO) enrichment analysis for predicted miRNA target genes was performed using the oil palm (*Elaeis guineensis*, EG11) whole genome gene set as the background population. GO annotations were retrieved from PLAZA (dicot/monocot plant-specific GO repository) combined with in-house oil palm genome annotation, non-plant-derived GO terms (e.g., animal synapse-related, viral-origin terms) were filtered prior to enrichment testing. Only plant relevant GO terms were retained for downstream visualization. *P*-values were adjusted by Benjamini-Hochberg FDR correction; terms with FDR q-value<0.05 were considered significantly enriched.

### 2.5 Quantitative real time PCR for determining miRNA and target transcript abundance

qPCR was used to examine the expression levels of selected miRNAs and their corresponding target genes. miRNA-derived cDNA was synthesized using a SYBR One Step PrimeScript miRNA cDNA Synthesis Kit (Takara, Otsu, Japan). qRT-PCR reactions were carried out on a Mastercycler ep realplex4 system. Each 10 μL reaction mixture contained 2 × SYBR Green qPCR ProMix (low ROX) (Biotech, Shanghai, China). The thermal cycling protocol consisted of initial denaturation at 95 °C for 5 seconds, followed by annealing at 55 °C for 15 seconds and extension at 68 °C for 20 seconds. Melting curve analysis was performed over a temperature gradient from 60 °C to 95 °C (20 minute ramp) to verify reaction specificity. All primer sequences, including miRNA specific forward qPCR primers, the kit-provided universal reverse primer and oligo-dT adaptor RT primer, the U6 housekeeping control primer for miRNA quantification, target-gene (*ATHB9-like*, *ATHB12-like*) qPCR primers, and Actin reference gene primers for target mRNA moralizations are listed in **Supplementary Table S1**. Three biological replicates (individual oil-palm seedlings) were used for each cold treatment, and each biological replicate was assayed in three technical qPCR replicates, yielding nine total measurements per miRNA/target transcript. Given this nested experimental structure (technical replicates nested within biological replicates), nested ANOVA with variance component decomposition was performed to separate total variation into biological-replicate variance and within biological technical replicate variance (**Supplementary Table S11**). Pairwise post-hoc comparisons were conducted using Tukey’s HSD test based on nested ANOVA outputs, with significance threshold set at *p* < 0.05. All qPCR statistical analyses were implemented in SPSS software.

Melting-curve analysis was performed from 60 °C to 95 °C with a continuous ramp. Representative melting-curve profiles for *Eg-miR393*, *Eg-miR166a* and U6 snRNA are presented in **Supplementary Figure S5**. Single sharp melting peaks were observed for all primer sets, confirming specific amplification and absence of primer dimer or off-target amplification products.

### 2.6 Time-series clustering of differentially expressed miRNAs

Time-series fuzzy c-means clustering was performed using the Mfuzz package (v2.6.0) in R (v4.2.1) for all differentially expressed miRNAs identified across the five cold stress time points.

The input matrix consisted of log_2_(fold-change) values (Cold vs. CK) at five time points (0.5, 1, 2, 4, and 8 h). Before clustering, expression profiles were standardized within each miRNA by z-score normalization to eliminate the influence of absolute expression magnitude and focus on temporal trend patterns. The optimal cluster number (c = 6) was determined based on the minimum centroid distance and partition coefficient. The fuzzification parameter m was set to 1.25. A membership value ≥ 0.7 was used as the threshold for assigning each miRNA to its corresponding cluster module; miRNAs with membership values below 0.7 were considered ambiguous and left unassigned. Cluster centroids were plotted to visualize the representative temporal expression patterns of each module.

### 2.7 Statistical analysis

All statistical evaluations were performed using SPSS software (Version 13, SPSS Inc., Chicago, IL, USA). For physiological measurements (chlorophyll, MDA, relative electrical conductivity, and CIELAB color indices), multiple technical measurements per biological replicate were averaged first. Group comparisons were performed using one-way ANOVA followed by Tukey’s HSD post-hoc test. For qPCR expression data featuring a nested design (technical replicates nested within biological replicates), nested ANOVA with variance-component decomposition was applied (**Supplementary Table S11**), followed by Tukey’s HSD post-hoc comparisons. For all tests, *p<* 0.05 was considered statistically significant. For small RNA sequencing data, differential expression analysis of miRNAs between the control (CK) and each cold treatment group was followed by Benjamini-Hochberg (BH) false discovery rate (FDR) correction applied independently within each comparison. miRNAs with an adjusted q-value< 0.05 and |log_2_ fold-change| ≥ 1 were considered significantly differentially expressed. Both raw *p*-values and BH-adjusted q-values are reported in **Supplementary Tables S3**–**S7**.

## 3 Results

### 3.1 Changes in chlorophyll, MDA, and electrical conductivity in oil palm leaves under cold stress

Under cold stress at 8 °C, chilling injury in oil palm leaves progressed in a clear time-dependent manner. No obvious phenotypic changes were observed after 0.5 h of treatment compared with the control (0 h). After 1 h, the leaves began to show slight yellowing. At 2 h, slight browning appeared, indicating the onset of the stress response. Following 4 h of treatment, large and severe brown lesions developed on the leaves. When the treatment was extended to 8 h, these brown lesions spread to nearly the entire leaf surface, indicating that the plants had suffered severe chilling injury (**Figure 1A**). To evaluate the physiological responses to cold stress, chlorophyll concentration, MDA concentration, and electrical conductivity were measured in the leaves of oil palm seedlings exposed to six different treatment durations (0, 0.5, 1, 2, 4, and 8 hours), alongside a control group (CK). Compared with the CK, chlorophyll a, chlorophyll b, and total chlorophyll concentrations showed a modest decrease under all stress treatments (**Figure 1B**). However, MDA concentration and leaf electrical conductivity exhibited a progressive, time-dependent increase with prolonged cold exposure across all cold stress durations and reached their maximum values at the 8-hour stress treatment group, where these values were significantly higher than those recorded for all other treatment groups (**Figures 1C, D**). Obvious visual leaf discoloration occurred along with chilling injury progression, therefore, quantitative colorimetric parameters were further analyzed to capture subtle and macroscopic color shifts. With the prolongation of cold exposure, a value increased gradually, reflecting enhanced red-brown pigmentation, while elevated *b* values indicated progressive leaf yellowing. The lightness parameter *L* decreased notably at later stress stages (4 h and 8 h), corresponding to leaf browning and loss of luster. Hue angle h° continuously declined, suggesting a color transition from healthy green toward yellow-brown tones. The total color difference *ΔE*_ab_, representing the magnitude of color divergence relative to non-stressed CK, kept increasing as cold stress duration extended. Even at 0.5 h of cold treatment when no obvious visual phenotypic alteration could be distinguished, a measurable *ΔE*_ab_ value was detected, and *ΔE*_ab_ reached its maximum at 8 h, consistent with the severe browning phenotype. Pearson correlation analysis revealed that *ΔE*_ab_ was significantly positively correlated with MDA concentration (*r* = 0.972, *p* < 0.001, n = 18) and relative electrical conductivity (*r* = 0.956, *p* < 0.001, n = 18), and significantly negatively correlated with total chlorophyll content (*r* = −0.941, *p* < 0.001, n = 18). These results demonstrated that total color-difference index could quantitatively mirror the severity of cold-induced physiological damage in oil palm seedlings. The color-difference data are presented in **Figure 1E**. The hue angle h°, representing leaf color tone, decreased progressively with prolonged cold stress exposure. The CK group exhibited high h° values (green-toned leaves). Upon cold treatment, h° gradually declined, reflecting the visual shift from healthy green towards yellow-brown discoloration under chilling injury. The full h° and chroma values for each treatment group are provided in **Supplementary Table S13**, including all CIELAB indices (*L*, a*, b*, h°, C, *ΔE*_ab_).

**Figure 1:**
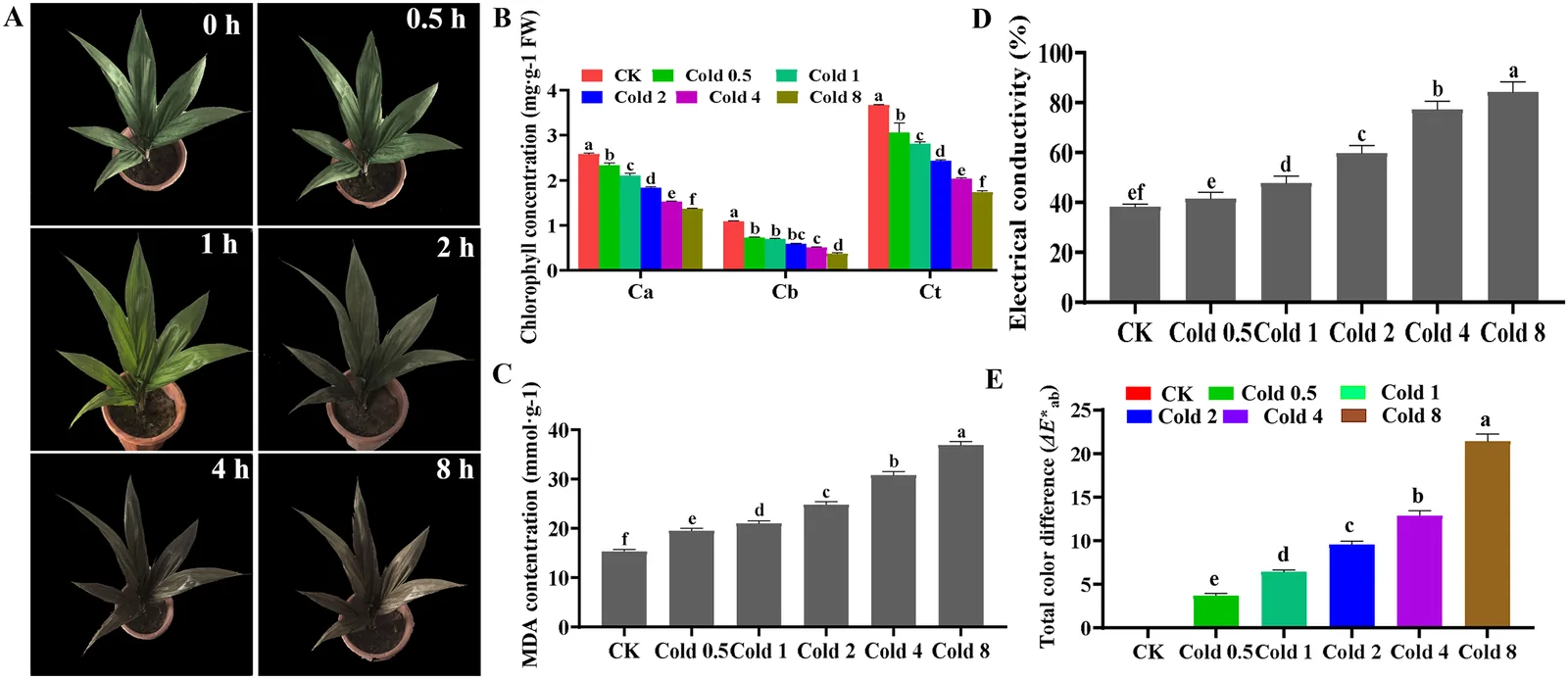
Physiological changes in oil palm (*Elaeis guineensis*) seedlings under different cold stress conditions. **(A)** Phenotypes of oil palm seedlings subjected to different cold treatment durations. **(B)** Contents of chlorophyll a (Ca), chlorophyll b (Cb), and total chlorophyll (Ct) in seedling leaves. **(C)** Malondialdehyde (MDA) concentration in seedling leaves. **(D)** Relative electrical conductivity of seedling leaves. **(E)** Total color difference (*ΔE*_ab_) representing the magnitude of color divergence relative to non-stressed CK, kept increasing as cold stress duration extended. For physiological assays, multiple technical measurements were conducted on each biological sample, technical replicates were averaged for each biological replicate prior to statistical analysis and plotting. Data represent means ± standard error of biological replicates (SE, n = 3 independent oil palm seedlings). Different lowercase letters above bars indicate significant differences according to Tukey’s post-hoc test (*p* < 0.05).

### 3.2 Construction and next-generation sequencing of small RNA libraries in oil palm

To identify miRNAs responsive to cold stress in oil palm, 18 independent small RNA cDNA libraries corresponding to 18 individual biological samples (three biological replicates for each of six experimental conditions: 0 h-control, 0.5 h, 1 h, 2 h, 4 h, and 8 h cold stress) were sequenced on the Illumina platform. A statistical summary of the sequencing data is provided in **Table 1**. After adapter trimming and quality filtering, an average of 14.30, 11.63, 9.70, 15.03, 13.74, and 13.19 million clean reads were obtained for the 0 h, 0.5 h, 1 h, 2 h, 4 h, and 8 h samples, respectively. The clean reads represented 98.76%, 98.84%, 98.81%, 98.93%, 99.13%, and 98.81% of the total raw reads across the six samples, confirming the high quality of the data for downstream analyses.

**Table 1:** Summary of small RNA sequencing results from oil palm plants under normal temperature (CK) and at 0.5, 1, 2, 4, and 8 hours after cold stress.

ID	Clean_reads	High_quality	3’adapter_null	Insert_null	5’adapter_Contaminants	PolyA	Clean_tags
CK-1	15951278 (100%)	15816811 (99.1570%)	23452 (0.1483%)	54648 (0.3455%)	13014 (0.0823%)	1492 (0.0094%)	15246587 (95.5822%)
CK-2	15120365 (100%)	14982435 (99.0878%)	15147 (0.1011%)	59387 (0.3964%)	12343 (0.0824%)	1483 (0.0099%)	14356989 (94.9513%)
CK-3	11831430 (100%)	11598451 (98.0308%)	286767 (2.4725%)	61665 (0.5317%)	9845 (0.0849%)	1399 (0.0121%)	10775669 (91.0766%)
Cold 0.5-1	10575714 (100%)	10429007 (98.6128%)	50924 (0.4883%)	75340 (0.7224%)	11809 (0.1132%)	1077 (0.0103%)	9886401 (93.4821%)
Cold 0.5-2	14404838 (100%)	14237580 (98.8389%)	19710 (0.1384%)	68666 (0.4823%)	18579 (0.1305%)	843 (0.0059%)	12769172 (88.6450%)
Cold 0.5-3	9900170 (100%)	9808403 (99.0731%)	10670 (0.1088%)	47964 (0.4890%)	9602 (0.0979%)	684 (0.0070%)	9254766 (93.4809%)
Cold 1-1	9695254 (100%)	9572354 (98.7324%)	20525 (0.2144%)	42689 (0.4460%)	67147 (0.7015%)	526 (0.0055%)	8914834 (91.9505%)
Cold 1-2	9283447 (100%)	9175594 (98.8382%)	96147 (1.0479%)	55702 (0.6071%)	8409 (0.0916%)	616 (0.0067%)	8622759 (92.8832%)
Cold 1-3	10114400 (100%)	9998594 (98.8550%)	21291 (0.2129%)	72484 (0.7249%)	14014 (0.1402%)	832 (0.0083%)	9378811 (92.7273%)
Cold 2-1	14164968 (100%)	13999590 (98.8325%)	40937 (0.2924%)	83448 (0.5961%)	14878 (0.1063%)	967 (0.0069%)	12915111 (91.1764%)
Cold 2-2	15315793 (100%)	15162916 (99.0018%)	23302 (0.1537%)	83899 (0.5533%)	15180 (0.1001%)	1170 (0.0077%)	14192299 (92.6645%)
Cold 2-3	15616520 (100%)	15453806 (98.9581%)	21775 (0.1409%)	59496 (0.3850%)	14013 (0.0907%)	889 (0.0058%)	14545648 (93.1427%)
Cold 4-1	15552268 (100%)	15438986 (99.2716%)	17492 (0.1133%)	63581 (0.4118%)	16630 (0.1077%)	871 (0.0056%)	14411740 (92.6665%)
Cold 4-2	16559223 (100%)	16417982 (99.1471%)	17194 (0.1047%)	81149 (0.4943%)	14972 (0.0912%)	996 (0.0061%)	15306258 (92.4334%)
Cold 4-3	9116760 (100%)	9021808 (98.9585%)	6581 (0.0729%)	57229 (0.6343%)	10600 (0.1175%)	476 (0.0053%)	8393582 (92.0676%)
Cold 8-1	13922454 (100%)	13750072 (98.7618%)	21685 (0.1577%)	50293 (0.3658%)	12310 (0.0895%)	838 (0.0061%)	12973431 (93.1835%)
Cold 8-2	13408987 (100%)	13235244 (98.7043%)	27519 (0.2079%)	51534 (0.3894%)	12560 (0.0949%)	913 (0.0069%)	12399843 (92.4741%)
Cold 8-3	12234430 (100%)	12108217 (98.9684%)	65219 (0.5386%)	64620 (0.5337%)	13106 (0.1082%)	590 (0.0049%)	11396266 (93.1491%)

As shown in **Figure 2A**, the length of oil palm small RNAs (sRNAs) was predominantly distributed between 21 and 25 nt. With prolonged cold stress, distinct trends in sRNA length distribution were observed. Compared with the CK control, the proportion of 21-nt sRNAs initially decreased (by 14.55% at 0.5 h and 5.46% at 1 h) and then increased (by 14.55% at 2 h, 14.55% at 4 h, and 3.64% at 8 h). The proportion of 22-nt sRNAs continuously increased, with relative increases of 6.67%, 3.33%, 23.33%, 16.67%, and 16.67% at 0.5, 1, 2, 4, and 8 h, respectively. The proportion of 23-nt sRNAs first decreased (by 6.56% at 0.5 h and 9.84% at 1 h) and then increased (by 3.28% at 2 h and 1.64% at 4 h), returning to the control level at 8 h. Conversely, the proportion of 24-nt sRNAs showed an overall decreasing trend, with reductions of 1.25%, 4.03%, 5.01%, 3.20%, and 1.53% across the five cold stress time points.

**Figure 2:**
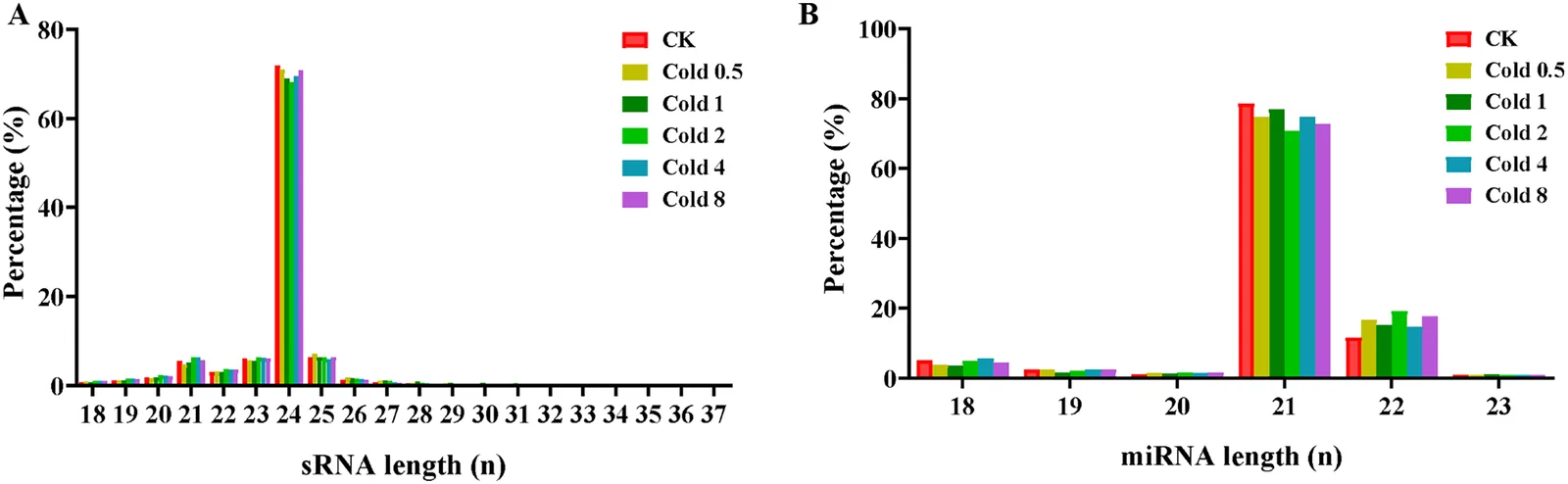
Small RNA **(A)** and miRNA **(B)** length distributions in oil palm under cold stress.

To further evaluate reproducibility and global expression patterns across the 18 small RNA sequencing libraries, we performed Pearson correlation analysis and principal component analysis (PCA) based on normalized miRNA read counts. The Pearson correlation heatmap showed high pairwise correlations among biological replicates within each treatment group (>0.95), confirming reliable sequencing reproducibility (**Supplementary Figure S1**). The CK control group exhibited low correlations with all cold treated samples (r = 0.18-0.24), indicating profound global reprogramming of miRNA expression upon cold exposure. Notably, samples harvested from 0.5 h to 8 h of cold treatment were highly correlated with one another, indicating that the global miRNA profile was rapidly reshaped within the first 0.5 h and remained relatively stable thereafter.

Consistent with the correlation results, PCA showed that the three CK replicates formed a distinct, tightly clustered group separated from all cold stressed samples along PC1, which explained 90.10% of total expression variance (**Supplementary Figure S2**). PC2 accounted for 7.1% of the variance and further discriminated cold samples collected at different time points. All biological replicates within each cold treatment group clustered closely. Although major miRNA transcriptional reprogramming occurred rapidly within 0.5 h after cold exposure, subtle time dependent expression shifts were still captured along PC2 across the 8 h cold stress period.

### 3.3 Identification and characterization of cold-responsive sRNAs in oil palm

By comparison with the precursor and mature miRNAs of all plants in the miRBase 22 database, an average of 4,897,597.67 (36.55%), 3,056,601.00 (28.14%), 2,988,541.33 (33.39%), 5,225,316.33 (37.73%), 4,963,336.33 (39.05%), and 4,288,933.00 (34.94%) unique sequences from the CK, Cold 0.5, Cold 1, Cold 2, Cold 4, and Cold 8 experimental groups, respectively, showed similarity to known miRNAs. In addition, based on annotations from the Rfam database, other types of unique sequences were also identified, including rRNA, snRNA, snoRNA, and tRNA. For rRNA, the average numbers and percentages of unique sequences were 635,676 (4.70%) at CK, 1,065,670.67 (10.07%) at Cold 0.5, 930,621.67 (10.36%) at Cold 1, 1,103,264 (7.93%) at Cold 2, 1,019,074.67 (7.96%) at Cold 4, and 1,084,469.33 (8.98%) at Cold 8. For snRNA, the average number of unique sequences were 976,833.33 (0.07%), 1,878,400.00 (0.17%), 1,299,900.00 (0.15%), 1,255,333.33 (0.09%), 2,613,000.00 (0.21%), and 2,012,333.33 (0.17%) for CK, Cold 0.5, Cold 1, Cold 2, Cold 4 and Cold 8 groups, respectively. The average numbers of unique snoRNA sequences were 7,477.33 (0.06%), 9,190.00 (0.08%), 7,911.33 (0.09%), 10,053.33 (0.07%), 10,719.00 (0.08%), and 9,254.00 (0.08%). For tRNA, the average number of unique sequences were 47,817.67 (0.36%), 78,242.67 (0.74%), 122,093.33 (1.34%), 111,027.33 (0.79%), 121,539.00 (0.99%), and 129,125.67 (1.04%) at CK, Cold 0.5, Cold 1, Cold 2, Cold 4, and Cold 8, respectively. The distribution of annotated sequences across the six experimental groups is summarized in **Table 2**.

**Table 2:** Distribution of unique small RNA (sRNA) sequences from oil palm plants under normal temperature (CK) and at 0.5, 1, 2, 4, and 8 hours after cold stress. “Unann” denotes sRNA sequences for which no annotation information could be obtained through database comparisons.

Sample	Total_abundance	rRNA_abundance	snRNA_abundance	snoRNA_abundance	tRNA_abundance	Exon_sense_abundance	Known_mirna_abundance	Novel_mirna_abundance
all	215740166	17516329 (8.12%)	301074 (0.14%)	163815 (0.08%)	1829537 (0.85%)	1614450 (0.75%)	76260977 (35.35%)	367475 (0.17%)
CK-1	15246587	817498 (5.36%)	13074 (0.09%)	9063 (0.06%)	49978 (0.33%)	97234 (0.64%)	5575194 (36.57%)	23868 (0.16%)
CK-2	14356989	588743 (4.10%)	8806 (0.06%)	7326 (0.05%)	54540 (0.38%)	95550 (0.67%)	4980806 (34.69%)	22910 (0.16%)
CK-3	10775669	500787 (4.65%)	7425 (0.07%)	6043 (0.06%)	38935 (0.36%)	70169 (0.65%)	4136793 (38.39%)	17183 (0.16%)
Cold 0.5-1	9886401	788943 (7.98%)	13110 (0.13%)	8083 (0.08%)	62645 (0.63%)	75775 (0.77%)	2906653 (29.40%)	23214 (0.23%)
Cold 0.5-2	12769172	1272652 (9.97%)	31618 (0.25%)	12810 (0.10%)	90685 (0.71%)	104069 (0.82%)	4256525 (33.33%)	27850 (0.22%)
Cold 0.5-3	9254766	1135417 (12.27%)	11624 (0.13%)	6677 (0.07%)	81398 (0.88%)	84061 (0.91%)	2006625 (21.68%)	15773 (0.17%)
Cold 1-1	8914834	854430 (9.58%)	7681 (0.09%)	6158 (0.07%)	55884 (0.63%)	64429 (0.72%)	3224897 (36.17%)	12959 (0.15%)
Cold 1-2	8622759	891653 (10.34%)	15960 (0.19%)	7194 (0.08%)	73854 (0.86%)	63007 (0.73%)	2994496 (34.73%)	13186 (0.15%)
Cold 1-3	9378811	1045782 (11.15%)	15356 (0.16%)	10382 (0.11%)	236542 (2.52%)	67483 (0.72%)	2746231 (29.28%)	13073 (0.14%)
Cold 2-1	12915111	952043 (7.37%)	11022 (0.09%)	8648 (0.07%)	76496 (0.59%)	92499 (0.72%)	5233728 (40.52%)	18130 (0.14%)
Cold 2-2	14192299	1219521 (8.59%)	11441 (0.08%)	10508 (0.07%)	103973 (0.73%)	110564 (0.78%)	5094969 (35.90%)	21678 (0.15%)
Cold 2-3	14545648	1138228 (7.83%)	15197 (0.10%)	11004 (0.08%)	152613 (1.05%)	109721 (0.75%)	5347252 (36.76%)	22960 (0.16%)
Cold 4-1	14411740	1131671 (7.85%)	31887 (0.22%)	12520 (0.09%)	133313 (0.93%)	106585 (0.74%)	5614588 (38.96%)	19936 (0.14%)
Cold 4-2	15306258	1281699 (8.37%)	24837 (0.16%)	12737 (0.08%)	135026 (0.88%)	114303 (0.75%)	6006254 (39.24%)	25844 (0.17%)
Cold 4-3	8393582	643854 (7.67%)	21666 (0.26%)	6900 (0.08%)	96278 (1.15%)	62027 (0.74%)	3269167 (38.95%)	11667 (0.14%)
Cold 8-1	12973431	854052 (6.58%)	15439 (0.12%)	7765 (0.06%)	170001 (1.31%)	105685 (0.81%)	4470358 (34.46%)	21332 (0.16%)
Cold 8-2	12399843	981568 (7.92%)	10468 (0.08%)	7831 (0.06%)	146147 (1.18%)	100455 (0.81%)	4668035 (37.65%)	20111 (0.16%)
Cold 8-3	11396266	1417788 (12.44%)	34463 (0.30%)	12166 (0.11%)	71229 (0.63%)	90834 (0.80%)	3728406 (32.72%)	35801 (0.31%)

### 3.4 Identification of conserved miRNAs under cold stress in oil palm

In oil palm (*Elaeis guineensis*), a total of 191 unfiltered known mature miRNA candidates were identified across six experimental groups (aggregated from 18 independent sequencing libraries). The average number of detected known-miRNA candidates per experimental group (averaged across three biological replicates) was as follows: 203 in CK, 181 in Cold 0.5, 200 in Cold 1, 214 in Cold 2, 249 in Cold 4, and 210 in Cold 8. No miRNA candidate was ubiquitously detected in every single biological replicate across all six experimental groups, a core set of 86 known miRNA candidates was detected in at least one biological replicate within each of the six experimental groups. The average number of predicted target genes for these miRNAs in the CK, Cold 0.5, Cold 1, Cold 2, Cold 4, and Cold 8 experimental groups was 2,982, 2,996, 3,936, 4,142, 4,517, and 4,663, respectively (**Table 3**). Note that the value of 191 refers to distinct mature miRNA sequences, whereas **Table 3** reports a total of 269 conserved miRNA entries when counting distinct miRNA isoforms/variants across all samples. In total, 6,179 target genes corresponding to these 269 conserved miRNA entries were obtained from the six treatment samples.

**Table 3:** Summarizes the predicted target gene counts for conserved and novel miRNAs, as well as the number of novel miRNA precursor candidates.

Sample	Conserved miRNAs no.	Average conserved miRNAs no.	Predicted target genes no. of conserved miRNAs	Average predicted target genes no. of conserved miRNAs	Novel miRNAs no.	Average novel miRNAs no.	Predicted target genes no. of novel miRNAs	Average predicted target genes no. of novel miRNAs
CK-1	202	203	2985	2982	43/33	42/32	2149	2162
CK-2	205		2977		41/31		2163	
CK-3	202		2984		42/32		2174	
Cold 0.5-1	181	181	2997	2996	72/48	71/47	1639	1645
Cold 0.5-2	176		2995		70/48		1646	
Cold 0.5-3	186		2996		72/46		1650	
Cold 1-1	198	200	3937	3936	54/42	55/41	3694	3703
Cold 1-2	203		3937		56/40		3682	
Cold 1-3	199		3934		55/42		3733	
Cold 2-1	213	214	4140	4142	65/47	65/46	2108	2098
Cold 2-2	222		4146		63/45		2105	
Cold 2-3	207		4140		68/46		2081	
Cold 4-1	252	249	4485	4517	76/52	75/51	1128	1125
Cold 4-2	249		4537		75/51		1122	
Cold 4-3	246		4529		75/50		1125	
Cold 8-1	211	210	4676	4663	89/61	90/62	2323	2323
Cold 8-2	205		4653		90/63		2328	
Cold 8-3	214		4660		92/64		2318	
Total	269	269	6179	6179	95/67	95/67	7978	7978

In the case of novel miRNAs, each value represents the total number identified, with the subsequent number denoting how many of these had predicted target gene loci.

After stringent filtering to remove 24-nt siRNA-derived sequences and animal origamis annotations, a total of 5,032 known mature plant-derived miRNAs were identified across six experimental groups. The average number of miRNAs per library was as follows: 514 in CK, 682 in Cold 0.5, 779 in Cold 1, 914 in Cold 2, 1,036 in Cold 4, and 1,107 in Cold 8. Notably, 286 miRNAs were common to all six experimental groups. The average number of predicted target genes for these miRNAs in the CK, Cold 0.5, Cold 1, Cold 2, Cold 4, and Cold 8 libraries was 2,982, 2,996, 3,936, 4,142, 4,517, 4,663, respectively (**Table 3**). In total, 2,937 target genes corresponding to the conserved miRNAs were obtained from the six treatment samples.

The length of filtered known oil palm miRNAs ranged from 18 to 23 nucleotides (nt), with 21-nt and 22-nt miRNAs representing the dominant size classes, consistent with canonical plant miRNA features. After filtering, 24-nt sequences were excluded from the known-miRNA dataset because they largely correspond to heterochromatic siRNAs. As shown in **Figure 2B**, the majority of miRNA sequences from the oil palm libraries began with uridine. These sequence characteristics are consistent with previously reported features of plant miRNAs (Czech and Hannon, 2011; Guo et al., 2017). Specifically, the proportions of 21-nt miRNAs in the CK, Cold 0.5, Cold 1, Cold 2, Cold 4, and Cold 8 experimental groups were 78.60%, 74.80%, 77.03%, 70.93%, 74.80%, and 72.90%, respectively, while those of 22-nt miRNAs were 11.60%, 16.70%, 15.27%, 19.27%, 14.70%, and 17.70%, respectively (**Figure 2B**). As shown in **Figure 3**, the majority of miRNA sequences from the oil palm libraries began with uridine. These sequence characteristics are consistent with previously reported features of plant miRNAs (Czech and Hannon, 2011; Guo et al., 2017). In oil palm, the percentages of adenine (A), uracil (U), guanine (G), and cytosine (C) at each position of the conserved miRNAs changed dynamically with increasing cold stress (**Figure 3**). Among these conserved miRNAs, one of the most abundant bases, G, was mainly present at positions 3, 4, 8, 9, 10, and 16, whereas U was predominantly distributed at positions 1, 12, 13, 16, and 17. Another highly abundant base, C, was primarily located at positions 6 and 19.

**Figure 3:**
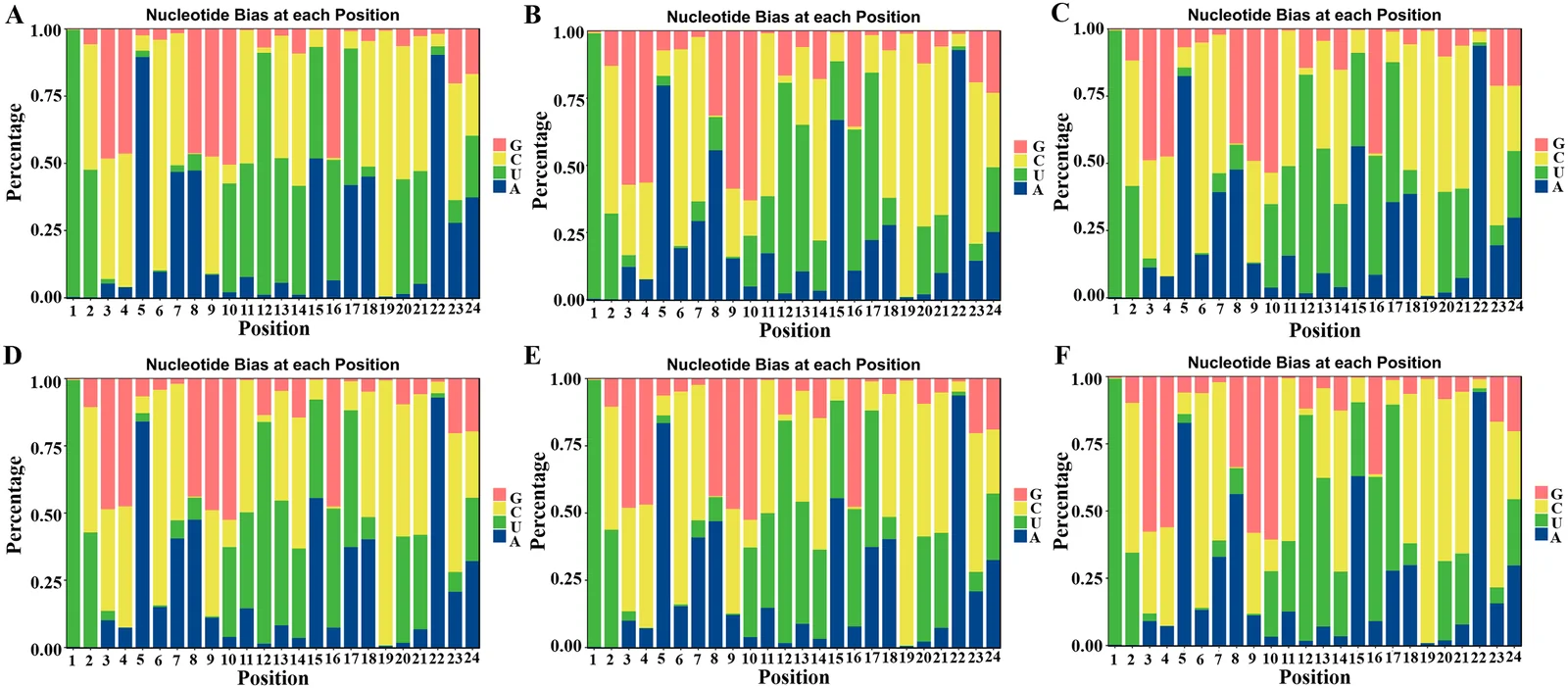
Nucleotide bias at each position of conserved miRNAs in oil palm under different cold stress conditions. The cold stress treatments were as follows: **(A)** CK; **(B)** Cold 0.5; **(C)** Cold 1; **(D)** Cold 2; **(E)** Cold 4; and **(F)**, Cold 8. The X-axis denotes the nucleotide position of the miRNA, and the Y-axis represents the percentage of each nucleotide at that position. Pink, yellow, blue, and green indicate guanine (G), cytosine (C), adenine (A), and uracil (U), respectively.

### 3.5 Identification of novel miRNAs in oil palm under cold stress

For novel miRNA identification, clean small-RNA reads were mapped to the oil palm reference genome (EG11, https://www.ncbi.nlm.nih.gov/datasets/genome/GCF_000442705.2/). Novel miRNAs were predicted primarily using miRDeep-P2 and filtered according to the plant-specific miRNA annotation criteria from Axtell and Meyers (2018), with miRDeep2, Mireap and miRdentify applied to provide auxiliary supporting evidence. After prediction and strict filtering, we obtained average novel miRNA counts of 42, 71, 55, 65, 75, and 90 for the CK, Cold 0.5, Cold 1, Cold 2, Cold 4, and Cold 8 experimental groups, respectively. In total, 95 distinct novel miRNA sequences were discovered across the six oil palm experimental groups. From these, putative target genes were inferred: specifically, an average of 2,162 targets from 32 novel miRNAs (CK), 1,645 from 47 (Cold 0.5), 3,703 from 41 (Cold 1), 2,098 from 46 (Cold 2), 1,125 from 51 (Cold 4), and 2,323 from 62 (Cold 8). Taken together, 7,978 target genes corresponding to 67 conserved novel miRNAs were recorded (**Table 3**).

As summarized in **Figure 4**, the length of novel miRNAs in each of the six experimental groups spanned 18–25 nt. Across all treatments, 21-nt species represented the dominant size class. Their read counts and frequency distributions were as follows: CK, 3,204 (44.46%); Cold 0.5, 5,036 (52.87%), Cold 1, 4,976 (50.95%), Cold 2, 4,358 (49.73%), Cold 4, 7,336 (46.12%), and Cold 8, 7,319 (51.80%). To expand the repertoire of known oil palm miRNAs, we turned to the oil palm transcriptome as a reference. The alignment coverage of transcriptome reads against the whole-genome assembly was quantified using HISAT (hierarchical indexing for spliced alignment of transcripts), applying the computational strategy outlined by Kim et al (Kim et al., 2015). From this approach, 995 miRNA sequencing reads were retrieved, enabling the identification of 1,207 putative target gene loci (**Supplementary Table S2**).

**Figure 4:**
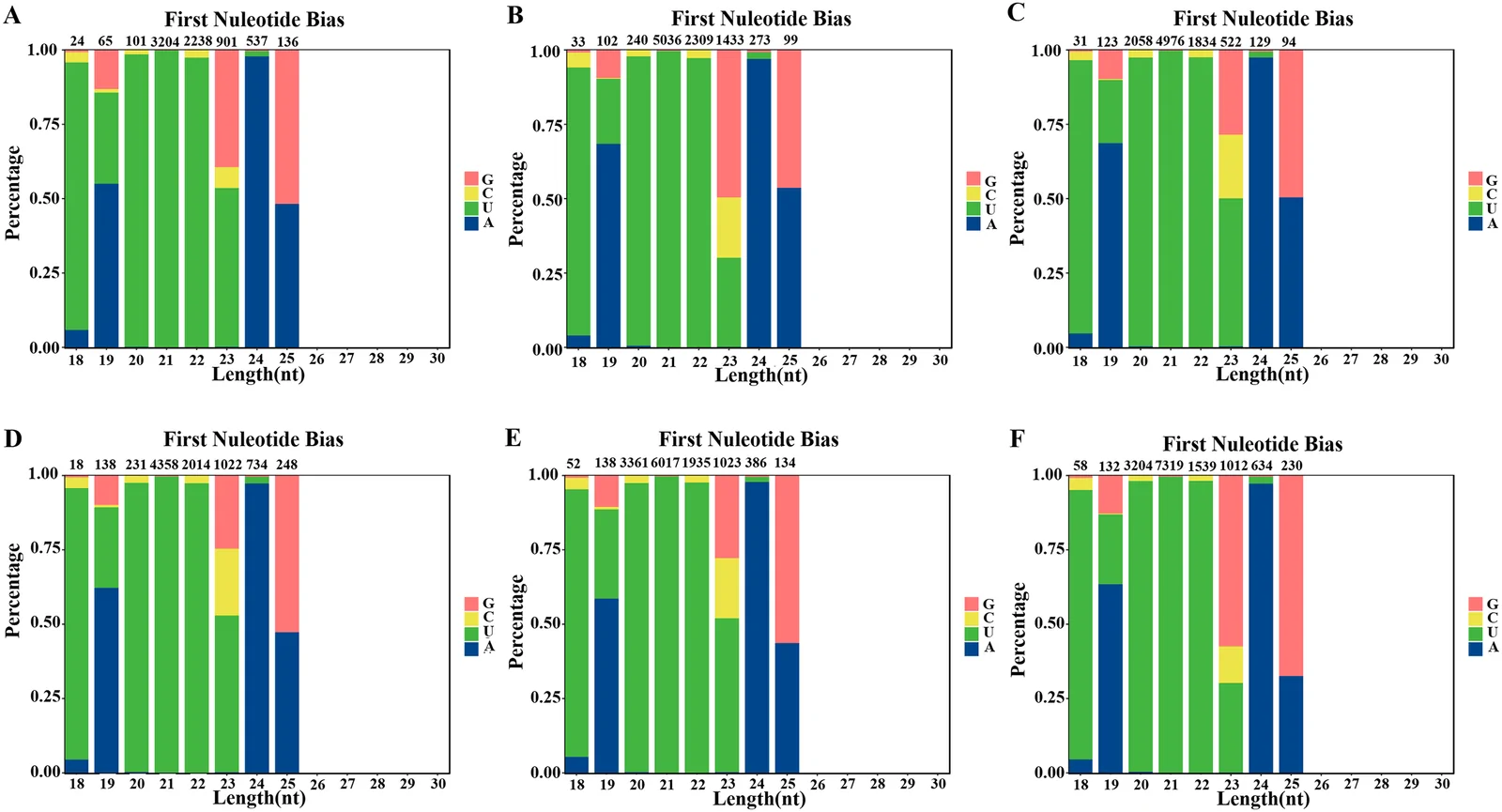
First nucleotide bias of length classes of high-confidence novel miRNAs in oil palm under different cold stress conditions. Novel miRNAs were predicted against *Elaeis guineensis* reference genome using miRDeep-P2 and filtered following plant-miRNA annotation criteria from Axtell and Meyers (2018). **(A)** CK, **(B)** Cold 0.5, **(C)** Cold 1, **(D)** Cold 2, **(E)** Cold 4, and **(F)** Cold 8. The X-axis represents the length classes of novel miRNAs (in nucleotides, nt), and the Y-axis indicates the percentage of each first nucleotide. Pink, yellow, blue, and green represent guanine (G), cytosine (C), adenine (A), and uracil (U), respectively.

### 3.6 Profiling of miRNA expression changes in oil palm under cold conditions

Under cold treatment conditions, the expression levels of miRNAs in oil palm (*Elaeis guineensis*) changed significantly (**Figure 5**). A total of 48 known mature miRNA sequences and 11 novel miRNAs were identified as differentially expressed via small RNA sequencing after strict plant specific miRNA annotation filtering.

**Figure 5:**
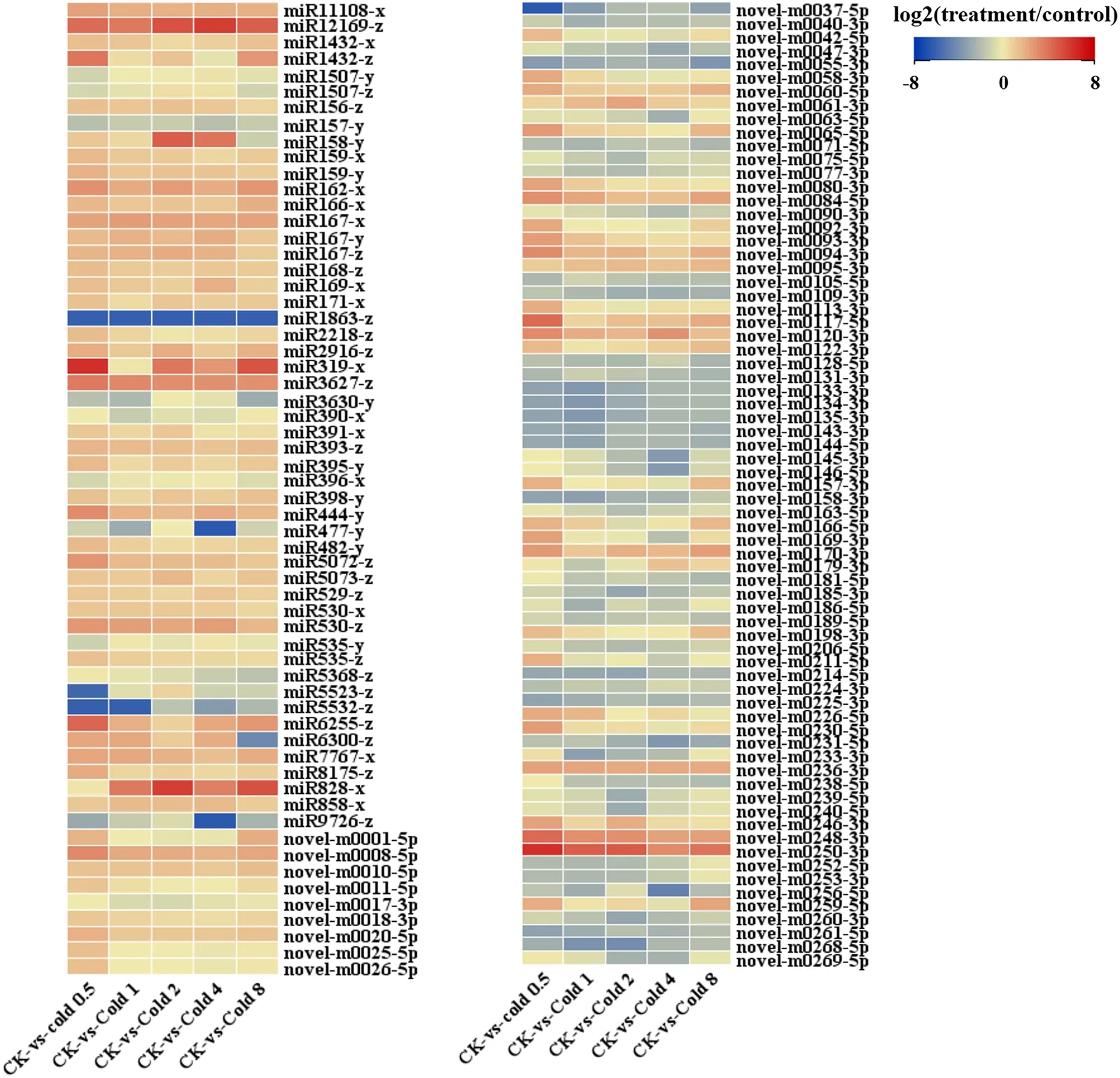
Heat map of differentially expressed conserved miRNAs in oil palm under CK, cold 0.5 h, cold 1 h, cold 2 h, cold 4 h and cold 8 h. The color scale represents relative miRNA expression levels, with varying hues indicating signal intensity. Red denotes up-regulation, and blue denotes down regulation of each miRNA under the respective stress conditions.

To investigate the effect of cold treatment duration on miRNA expression, we compared the miRNA expression profiles between the control group (CK) and five treatment time points: Cold 0.5, Cold 1, Cold 2, Cold 4, and Cold 8. The number of differentially expressed known miRNAs varied with treatment duration. Specifically, compared with CK, 32 differentially expressed miRNAs were detected under Cold 0.5 treatment, including 23 up-regulated and 9 down-regulated (**Supplementary Table S3**). Under Cold 1 treatment, 20 differentially expressed miRNAs were identified (16 up-regulated and 4 down-regulated, **Supplementary Table S4**). Under Cold 2 treatment, 24 differentially expressed miRNAs were identified (22 up-regulated and 2 down-regulated, **Supplementary Table S5**). Similarly, Cold 4 treatment also resulted in 24 differentially expressed miRNAs (20 up-regulated and 4 down-regulated, **Supplementary Table S6**). Under Cold 8 treatment, the number decreased to 17 differentially expressed miRNAs (11 up-regulated and 6 down-regulated, **Supplementary Table S7**). Overall, as cold treatment duration increased, the number of differentially expressed miRNAs first increased and then decreased. Higher numbers were observed under Cold 0.5, Cold 2, and Cold 4 treatments, followed by a decline under Cold 8 treatment. Notably, at the early-to-mid cold-stress time-points (0.5 h, 1 h, 2 h and 4 h), the number of up-regulated miRNAs exceeded the number of down-regulated miRNAs. At 8 h the counts were more comparable. These observations suggest that up-regulation represents the predominant response of miRNA expression during early and intermediate cold-stress exposure in oil palm.

To define the core and stage specific cold-responsive miRNA repertoires, we performed an intersection (Upset) analysis of differentially expressed miRNAs across the five cold treatment comparisons (CK vs. Cold 0.5/1/2/4/8 h), with separate panels for conserved and novel miRNAs (**Supplementary Figure S6**). Among conserved (known) miRNAs, nine were differentially expressed in all five comparisons and thus constitute the core cold responsive conserved set, comprising the up-regulated miR167-x, miR162-x, miR11108-x, miR3627-z, miR393-z, miR444-y, and miR7767-x, together with the down-regulated miR157-y and miR1863-z. In addition, 12, 2, 4, 5, and 2 conserved miRNAs were differentially expressed exclusively at Cold 0.5, 1, 2, 4, and 8 h, respectively, defining stage specific conserved responders, with the largest number of unique responders at the earliest (0.5 h) time point. Among novel miRNAs, 42 were differentially expressed across all five comparisons, defining the core novel cold responsive set, whereas 16, 8, 9, 8, and 8 novel miRNAs were uniquely differentially expressed at Cold 0.5, 1, 2, 4, and 8 h, respectively. The cold response of oil palm therefore comprises a shared core of miRNAs modulated throughout the stress time course, overlaid with stage specific sets induced or repressed only at particular treatment durations. The full membership of the core and stage specific sets is listed in **Supplementary Table S12**.

### 3.7 Gene ontology and Kyoto encyclopedia of genes and genomes pathway analyses of cold responsive miRNAs in oil palm

To investigate the functional distribution of differentially expressed genes in oil palm under different cold treatment durations, GO functional annotation and classification were performed on the unigenes from each experimental group. The total number of GO annotation assignments (nonunique GO term records, one gene may receive multiple GO terms) for predicted miRNA target genes were 30,762, 21,617, 21,707, 43,837, 29,145, and 29,701 for CK, Cold 0.5, Cold 1, Cold 2, Cold 4, and Cold 8 experimental groups, respectively (**Supplementary Table S8**). Note that these values represent total annotation instances rather than unique target gene count, individual genes can be annotated to multiple GO functional categories. The annotation results were categorized into three main GO classes: Biological Process, Cellular Component, and Molecular Function (**Figure 6**; **Supplementary Figure S1**; **Supplementary Table S8**).

**Figure 6:**
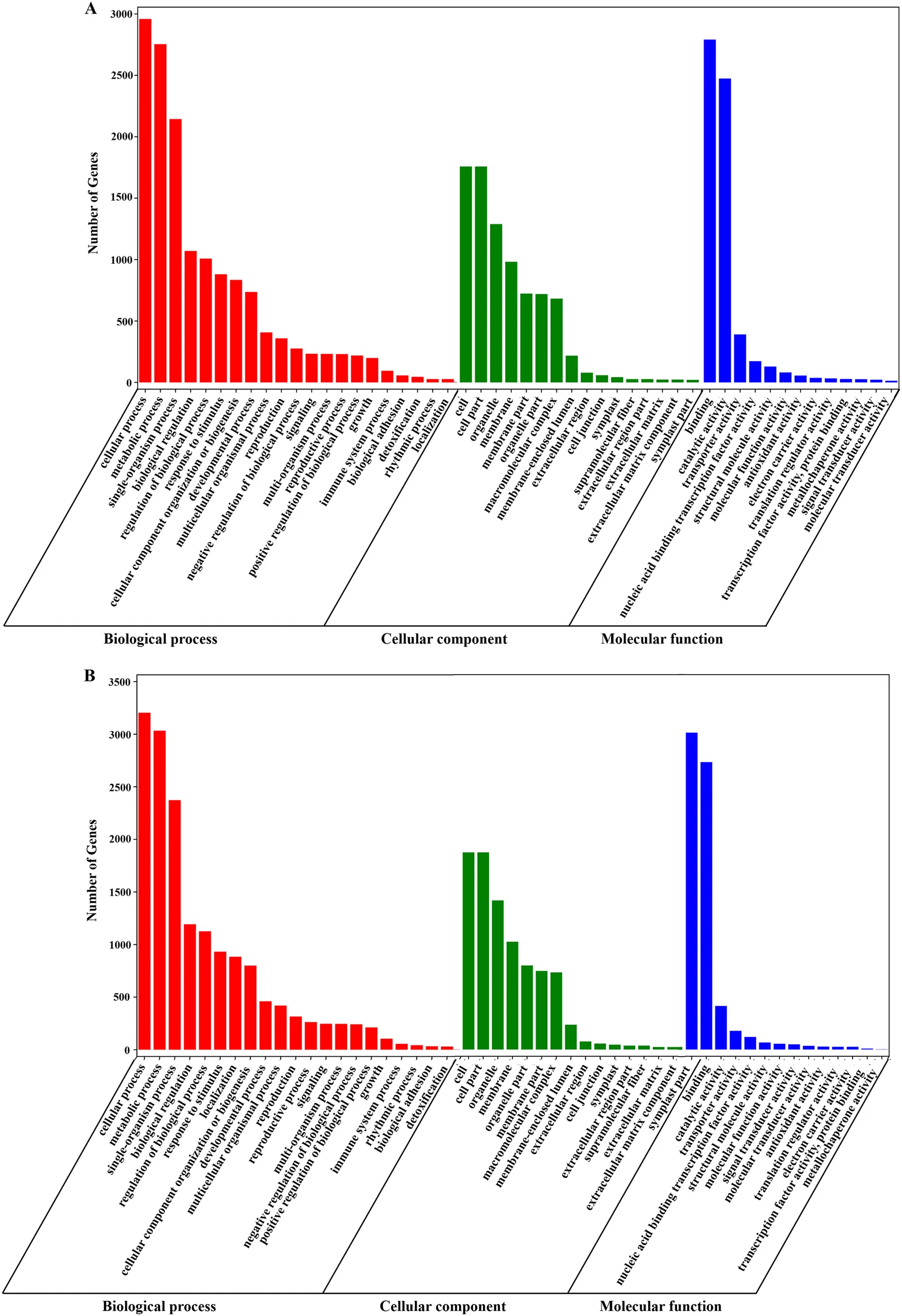
Gene ontology classification of putative target genes identified under cold stress conditions. **(A)** Control (CK, normal temperature), **(B)** Severe cold stress (8 hours).

Through KEGG enrichment analysis of miRNA target genes in oil palm samples subjected to cold treatment for different durations (0.5, 1, 2, 4, and 8 hours), this study revealed the dynamic changes in metabolic and signaling pathways in response to cold stress. The results showed that five pathways exhibited relatively high enrichment levels across various time points (**Figure 7**; **Supplementary Table S9**; **Supplementary Table S10**). Among these, the “Sesquiterpenoid and triterpenoid biosynthesis” pathway was significantly enriched (FDR-adjusted q< 0.05) across all five cold stress comparison groups (CK vs. Cold 0.5/1/2/4/8). According to Rich-Factor enrichment scores (**Supplementary Table S10**), this pathway exhibited the largest Rich-Factor value at 8 h of cold treatment, indicating the strongest enrichment at the late stress stage. This pathway was already detectable at early stress time point (0.5–2 h) and showed progressive strengthening under prolonged cold exposure, pointing to a sustained stress responsive role of terpenoid related metabolism in oil palm cold stress response. Functionally, sesquiterpenoids and triterpenoids are involved in phytohormone synthesis and cell membrane composition, with some sesquiterpenoid compounds also associated with plant stress defense. Under cold conditions, oil palm may adapt to environmental changes by regulating terpenoid metabolism.

**Figure 7:**
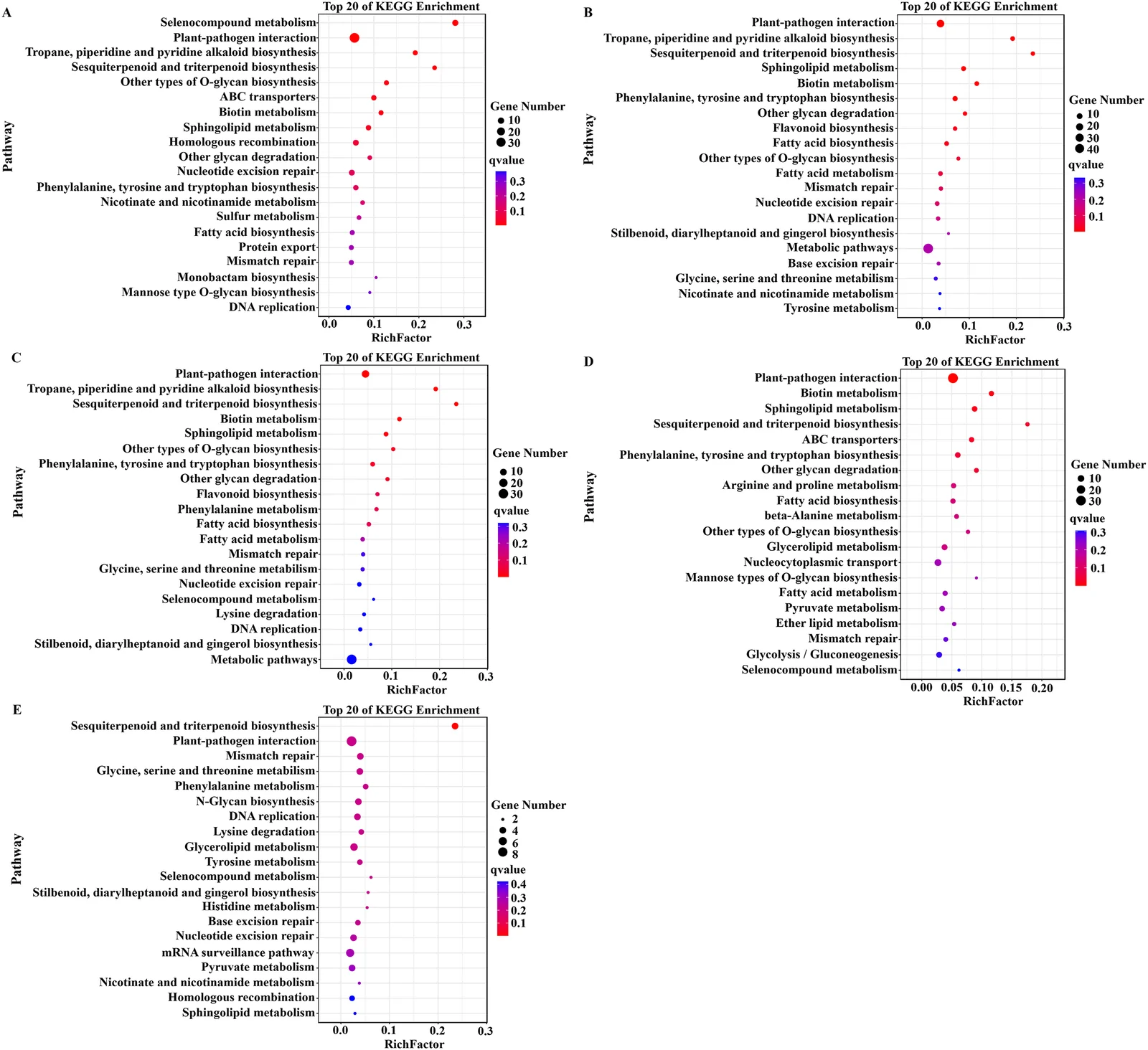
KEGG pathway enrichment analysis of differentially expressed conserved miRNA targets under cold stress. **(A)** CK vs. Cold 0.5, **(B)** CK vs. Cold 1, **(C)** CK vs. Cold 2, **(D)** CK vs. Cold 4, and **(E)** CK vs. Cold 8. X-axis represents Rich-Factor (enrichment score), bubble size corresponds to the numberof target genes assigned to each pathway, bubble color gradient corresponds to FDR-adjusted q-value (smaller q-value = higher significance). Y-axis shows enriched pathway names. Full enrichment statistics including raw p-value, FDR-q-value and Rich-Factor are provided in **Supplementary Table S10**.

During the early stage of stress (0.5–2 hours), the “Plant-pathogen interaction” pathway showed relatively high enrichment in the CK vs. Cold 1, 2, and 4 groups, suggesting potential overlap in signaling mechanisms between cold stress and pathogen infection. The “Tropane, piperidine and pyridine alkaloid biosynthesis” pathway was also significantly enriched at 0.5–2 hours, indicating that oil palm activates related chemical defense metabolism under low temperature. Moreover, the “Selen compound metabolism” pathway ranked first exclusively in the CK vs. Cold 0.5 group, implying that oil palm initiates antioxidant-related metabolism at the very early stage of cold exposure. During the middle stage (2–4 hours), the “Biotin metabolism” and “Sphingolipid metabolism” pathways were continuously enriched, suggesting that oil palm adjusts membrane system maintenance and energy metabolism. The “ABC transporters” pathway appeared enriched in the CK vs. Cold 4 group, which may be associated with transport functions (**Supplementary Figure S3**). During the late stage (8 hours), in addition to the terpenoid biosynthesis pathway, the “Mismatch repair” pathway also showed enrichment, suggesting that low temperature may cause certain DNA damage, thereby triggering repair mechanisms. The enrichment of the “Glycine, serine and threonine metabolism” and “Phenylalanine metabolism” pathways is related to amino acid metabolism and secondary metabolism. In summary, this study reveals the temporal response patterns of miRNA target gene pathways in oil palm under cold stress: the early stage primarily involves antioxidant and defense-related metabolism, the middle stage involves adjustments in membrane systems and energy metabolism, and the late stage involves responses such as DNA repair. Among these, the Sesquiterpenoid and triterpenoid biosynthesis pathway, which is enriched at all time points, represents a key metabolic pathway in the cold stress response of oil palm. These findings provide molecular level insights into the cold adaptability of oil palm.

**Figure 8** presents the enrichment of the Sesquiterpenoid and triterpenoid biosynthesis pathway, which was significantly enriched in all comparison groups: CK vs. Cold 0.5, CK vs. Cold 1, CK vs. Cold 2, CK vs. Cold 4, and CK vs. Cold 8. Multiple target genes of differentially expressed miRNAs were predicted to be enriched in this pathway. Among them, the gene encoding 3-hydroxy-3-methylglutaryl-CoA reductase (HMGCR, EC 1.1.1.354) was targeted by miR159 and miR396. This enzyme is a rate-limiting enzyme in the mevalonate (MVA) pathway and is involved in sesquiterpenoid and triterpenoid biosynthesis. The squalene synthase (SQS, EC 2.5.1.21) gene, targeted by miR172, participates in the synthesis of linear terpene precursors and the generation of squalene. Previous studies have shown that HMGCR is up-regulated in plants in response to oxidative stresses, thereby promoting the accumulation of terpenoid secondary metabolites (Zhang et al., 2020). In the present study, the above target genes exhibited differential expression patterns across different time points of cold treatment.

**Figure 8:**
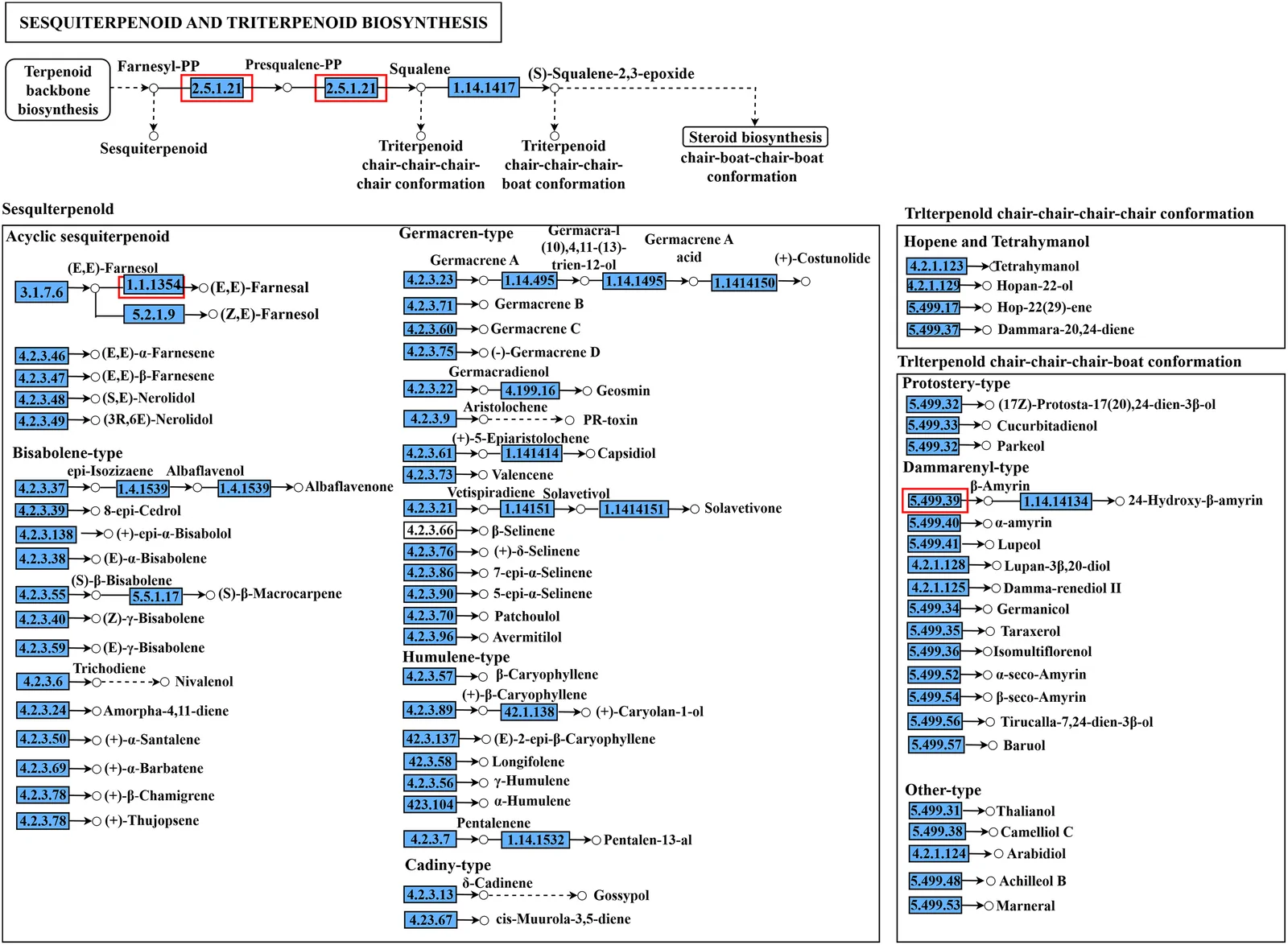
Schematic representation of the sesquiterpenoid and triterpenoid biosynthesis pathway. Pathway reaction information and EC numbers were obtained from the KEGG database (Kanehisa et al., 2025). Enzymes targeted by cold responsive miRNAs identified in this study are highlighted in red boxes. Solid arrows represent direct enzymatic reactions; dashed arrows indicate multi-step indirect connections.

Additionally, multiple target genes associated with downstream pathways of sesquiterpenoid and triterpenoid biosynthesis were identified. Among these, the β-amyrin synthase (β-AS, EC 5.4.99.39) gene, targeted by miR396, catalyzes the cyclization of 2,3-oxidosqualene to generate pentacyclic triterpene precursors. Previous studies have also demonstrated that terpene synthase genes are significantly induced in plants under cold stress, and their products are associated with membrane stability maintenance and signal transduction (Zhang et al., 2022; Gil et al., 2012). Collectively, these results show the enrichment of differentially expressed miRNA target genes in the sesquiterpenoid and triterpenoid biosynthesis pathway and their differential expression during cold treatment of oil palm.

### 3.8 miRNA expression profiles in oil palm under cold conditions

To validate the high-throughput sequencing results, 17 cold responsive plant miRNAs were selected for qPCR analysis. The selected miRNAs were Eg-miR156, Eg-miR157, Eg-miR159a, Eg-miR393, Eg-miR1863, Eg-miR9726, Eg-miR168, Eg-miR444, Eg-miR529, Eg-miR6255, Eg-miR630, Eg-miR167, Eg-miR166a, Eg-miR166-x, Eg-miR391-x, Eg-miR395-z, and Eg-miR398-y. qPCR analysis confirmed that all selected miRNAs were detectable in each treatment sample. Based on their expression changes between CK and different cold treatment time points (Cold 0.5, Cold 1, Cold 2, Cold 4, and Cold 8), these miRNAs were divided into two groups: Group 1, which exhibited higher expression levels than CK at all treatment time points (**Figure 9A**), and Group 2, which exhibited lower expression levels than CK at all treatment time points (**Figure 9B**).

**Figure 9:**
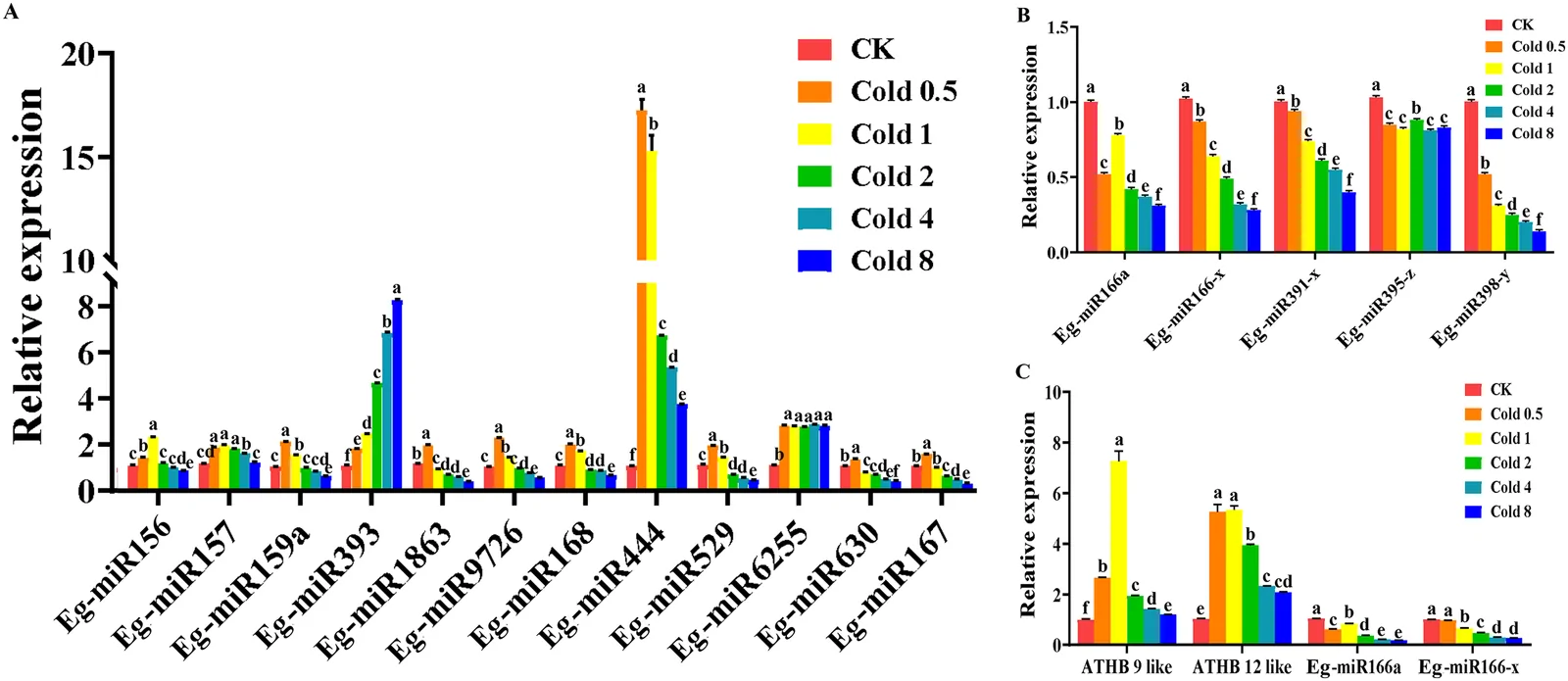
Expression of 17 miRNAs under CK, Cold 0.5 h, Cold 1 h, Cold 2 h, Cold 4 h, and Cold 8 h cold stress conditions. **(A)** miRNAs with higher expression levels than CK across all cold treatment time points. **(B)** miRNAs with lower expression levels than CK across all cold treatment time points. **(C)** Expression of miR166s and their computationally predicted target genes *ATHB9-like* and *ATHB12-like*. Relative expression of miRNAs was normalized to *U6* snRNA, relative transcript abundance of target mRNAs was normalized to *Actin*. Each biological replicate was measured in three nested technical qPCR replicates. Error bars represent standard error of biological replicates (n = 3 independent seedlings). Statistical significance was assessed via nested ANOVA followed by Tukey’s HSD post-hoc test. Different lowercase letters denote statistically significant differences (*p* < 0.05) determined by nested ANOVA followed by Tukey’s *post hoc* test.

Group 1 consisted of 12 miRNAs (**Figure 9A**), among which Eg-miR393 and Eg-miR444 were up-regulated under cold stress. The expression level of Eg-miR393 showed a linear increasing trend across all samples, with significant differences among treatments. The expression level of Eg-miR444 was significantly higher at Cold 0.5 than in CK, then gradually decreased at Cold 1, Cold 2, Cold 4, and Cold 8, but remained significantly different from CK. The remaining 12 miRNAs showed differential expression across treatment time points without a consistent up-regulation or down-regulation pattern. Group 2 contained five miRNAs: Eg-miR166a, Eg-miR166-x, Eg-miR391-x, Eg-miR395-z, and Eg-miR398-y (**Figure 9B**). The expression levels of all these miRNAs were lower than those in CK during cold stress, with the lowest levels observed at Cold 8. Among these, miR166 has been reported as a highly conserved stress responsive miRNA and is involved in root tip development (Li et al., 2022; Zhang et al., 2026). The negative expression relationship between miR166 members and their predicted target genes *ATHB9-like* and *ATHB12-like* only reflects transcript level abundance correlation from qPCR data. This correlation does not provide biochemical proof of miRNA-directed transcript cleavage, and degradome sequencing or 5′-RLM-RACE would be required to confirm physical target-cleavage events.

To verify the reliability of the small RNA-seq expression data, we performed qRT-PCR validation for selected cold responsive miRNAs across multiple cold stress time points. The log_2_(fold-change) values derived from RNA-seq and qRT-PCR exhibited strong correlations at all tested time points (**Supplementary Figure S4**), with *R^2^* values ranging from 0.9441 to 0.9960 (all *p* < 0.0001). These results demonstrated high consistency between qRT-PCR quantification and small RNA-seq transcriptome data, confirming the robustness of our sequencing datasets.

### 3.9 Stage-specific miRNAs in oil palm in response to cold stress

Based on our results, we constructed a diagram illustrating the phase-specific miRNAs expressed in oil palm under cold stress (**Figure 10**). Under normal temperature conditions (CK), oil palm plants exhibited normal growth and development, with electrical conductivity, chlorophyll concentration, and malondialdehyde (MDA) concentration all within normal ranges. Notably, four miRNAs (miR166a, miR166-x, miR395-z, and miR398-y) showed significantly higher expression levels in CK than in all cold treatment groups (Cold 0.5, Cold 1, Cold 2, Cold 4, and Cold 8).

**Figure 10:**
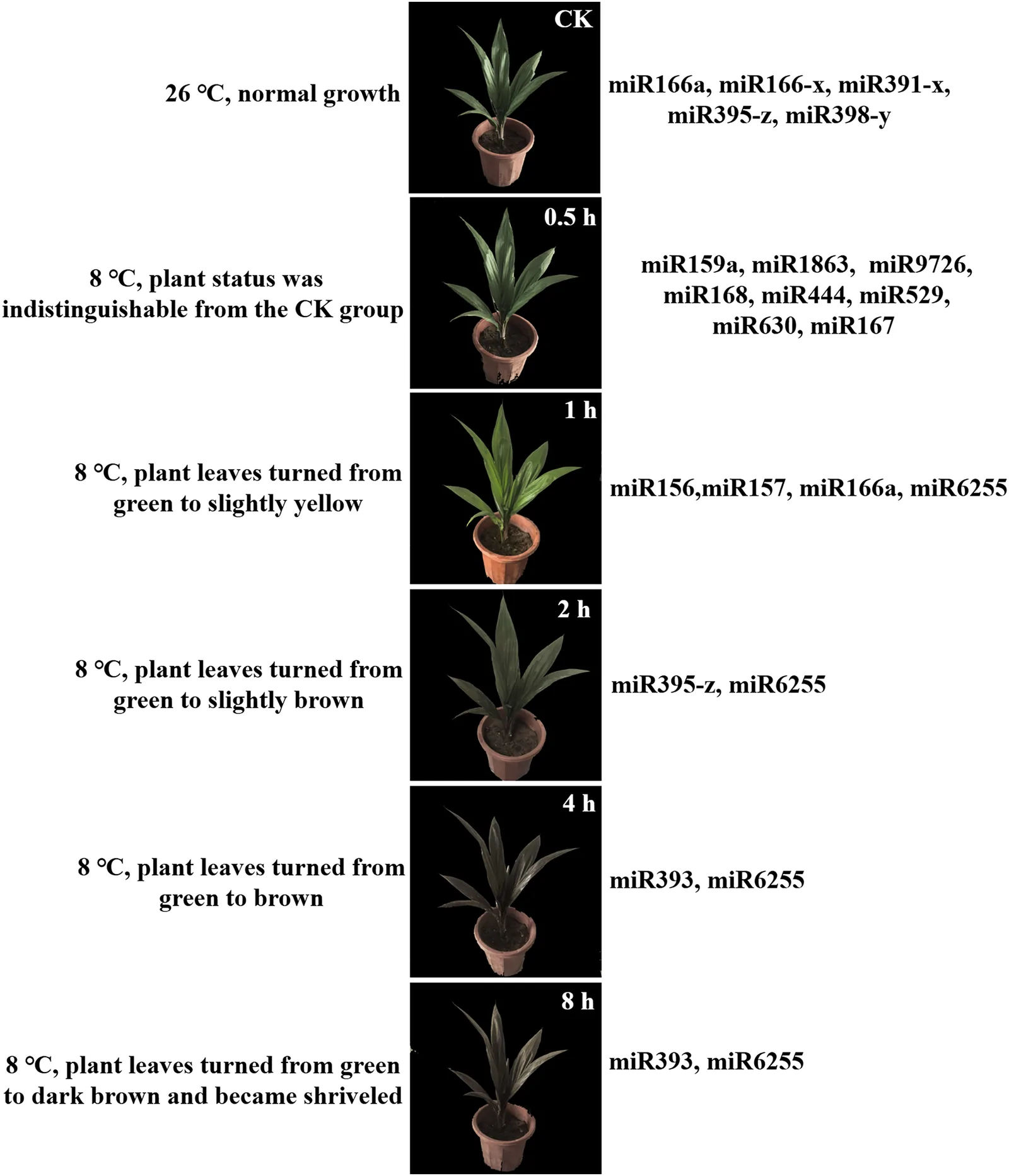
Phase-specific temporal response patterns of qPCR-validated cold-responsive miRNAs under cold stress at 8 °C. miRNAs are categorized manually into early-, intermediate-, late-response, sustained-up and sustained-down groups based on their measured expression trajectories across the time-course. This manual stage assignment is complemented by an unbiased genome wide Mfuzz clustering of all cold responsive miRNAs into six temporal modules.

Following 0.5 h of treatment at 8 °C, the appearance of oil palm leaves remained largely unchanged compared with the control group. However, chlorophyll (chlorophyll a, chlorophyll b, and total chlorophyll) concentrations decreased, and MDA concentration increased slightly, whereas leaf electrical conductivity was barely affected. At this stage, ten miRNAs (miR159a, miR1863, miR9726, miR168, miR444, miR529, miR630, and miR167) reached their maximum expression levels. When the treatment lasted for 1 h, the leaves began to turn yellow, accompanied by a decrease in chlorophyll concentration and increases in both leaf electrical conductivity and MDA concentration. These changes reflected a reduction in photosynthetic electron transfer capacity. At this stage, miR156, miR157, miR166a, and miR6255 showed peak expression. By 2 h of treatment, the leaves exhibited slight browning. Chlorophyll concentration continued to decline, while leaf MDA concentration and electrical conductivity increased significantly. The miRNAs with the highest expression levels at this stage were miR395-z and miR6255. After 4 h of treatment at 8 °C, extensive browning was observed on the leaves. Chlorophyll concentration continued to decrease, and leaf MDA concentration and electrical conductivity remained significantly elevated. At this stage, miR393 and miR6255 were the most highly expressed miRNAs. Upon 8 h of treatment, the leaves turned dark brown, lost their luster, and showed shrinkage at the margins. Chlorophyll concentration continued to decreased, while leaf MDA concentration and electrical conductivity increased further and more markedly. This substantial increase in cellular electrolyte leakage indicated a disruption of plant cell structure and functional integrity, ultimately affecting plant growth and development. At this final stage, miR393 and miR6255 remained the miRNAs with the highest expression levels. Based on qPCR-validated expression profiles of the 19 cold-responsive miRNAs across the complete cold stress time course (0 h, 0.5 h, 1 h, 2 h, 4 h, 8 h), we manually grouped these miRNAs into distinct temporal response patterns: early-transient response (peak at 0.5 h), intermediate-time response (peak at 1 h), late-sustained response (peak at 4–8 h), sustained up-regulation, and sustained down-regulation (summarized in **Figure 10**). This pairwise manual classification was complemented by an unbiased genome-wide fuzzy c-means time-series clustering (Kumar and Futschik, 2007) performed on the standardized log_2_ (fold-change) profiles of all 317 cold responsive miRNAs (266 novel and 51 conserved) across the five cold-treatment time points. Six distinct temporal modules were resolved (**Supplementary Figure S7**): M1 (69 miRNAs), an early-transient up-regulation peaking at 0.5 h, M2 (54 miRNAs), an early up-regulation followed by mid-phase (2 h) repression, M3 (37 miRNAs), an early-to-mid up-regulation that attenuates by 8 h, M4 (52 miRNAs), a mid-phase (4 h) repression with a late rebound, M5 (58 miRNAs), an early repression with intermediate (1–2 h) recovery, and M6 (47 miRNAs), a sustained down regulation across the time course. The early transient module M1 included the conserved early responsive members such as miR6255-z and miR168-z, corroborating their early induction in both sequencing and qPCR analyses.

## 4 Discussion

### 4.1 Changes in chlorophyll, MDA, and electrical conductivity in oil palm leaves under cold stress

Under 8 °C cold treatment, oil palm, a chilling-sensitive tropical palm, displayed progressive physiological damage with increasing stress duration. At the early stage (0.5-1h), visible leaf phenotypes remained largely normal, yet measurable shifts in CIELAB color parameters (elevated *ΔE*_ab_) were already detectable, prior to macroscopic yellowing or browning. This color divergence is not merely a visual descriptor: the CIELAB indices directly report underlying biochemical processes (Bacci et al., 1998). The reduction of lightness value (*L*) arises from chlorophyll degradation and loss of leaf glossiness, increases in a (red-green axis) and b (yellow-blue axis) correspond to accumulation of oxidized phenolic and carotenoid pigments driven by membrane lipid peroxidation (Gu et al., 2021). Accordingly, *ΔE*_ab_ showed strong positive correlation with MDA content and electrolyte leakage, and negative correlation with total chlorophyll, supporting that total color difference can serve as a high throughput proxy for tracking both chlorophyll breakdown and membrane oxidative injury in oil palm seedlings.

Membrane integrity was comparatively preserved within 0.5–1 h cold exposure, reflected by moderate MDA and low electrolyte leakage, representing the early perception-response phase. Starting from 2 h onward, MDA and relative electrical conductivity rose sharply, indicating massive membrane lipid peroxidation and loss of cellular compartmentation (Kumar et al., 2023). By 4–8 h, extensive leaf browning and tissue deterioration were observed. A crucial interpretive distinction must be made for these later time points: molecular changes observed at 4 h and 8 h are unlikely to represent purely adaptive cold acclimation programs. For this cold sensitive tropical species which cannot tolerate prolonged low temperature exposure, late-stage molecular profiles reflect a composite signal: residual stress adaptive reprogramming superimposed on large scale transcript perturbation associated with oxidative damage and incipient cell death (Imran et al., 2026). Physiologically, our data imply that the 2 h time point represents a physiological tipping point, beyond which chilling injury becomes largely irreversible. Integrating CIELAB pigment membrane readouts, our physiological measurements establish a clear temporal physiological trajectory: early perception (0.5 h), onset of membrane injury (1–2 h), and severe chilling damage/incipient cell deterioration (4–8 h).

### 4.2 Construction of oil palm small RNA libraries, sequencing analysis, and identification of cold responsive sRNAs

High-quality small RNA sequencing data was obtained across all 18 libraries, allowing us to probe sRNA population shifts accompanying cold stress progression. Across plant species, 24-nt sRNAs are predominantly heterochromatic siRNAs mediating RNA directed DNA methylation (RdDM), whereas 21-nt sRNAs encompass canonical plant miRNAs as well as phasiRNA species (Bartel, 2004; Matzke and Mosher, 2014). In oil palm, we observed a general reduction in the proportion of 24-nt sRNAs under cold stress, while 21-nt sRNA fractions declined transiently at 0.5-1h then rebounded a later time points. This pattern suggests cold stress perturbs heterochromatic siRNA homeostasis and remodels the pool of post transcription regulatory 21-nt small RNAs. Such remodeling occurs extremely rapidly, as supported by PCA and correlation analysis showing global miRNA profiles were already substantially rewired at the earliest 0.5 h time point. A fraction of rRNA derived fragments was present in our libraries, a commonly observed feature in plant small RNA sequencing and does not compromise downstream miRNA analysis (Lu et al., 2005). Collectively, our small RNA dataset captures the rapid and dynamic reprogramming of small RNA transcriptome in oil palm upon cold challenge.

### 4.3 Identification of conserved and novel miRNAs in oil palm under cold stress

We identified both conserved known miRNAs and high confidence novel miRNAs following strict plant miRNA annotation criteria (Axtell and Meyers, 2018). The number of detected novel miRNA candidates increased as cold stress persisted. This trend echoes observations in other crop species under abiotic stress (Guo et al., 2017; Thiebaut et al., 2014) and suggests species specific novel miRNAs constitute an important component of oil palm cold stress regulatory networks, beyond the well conserved miRNA families. Canonical plant miRNA features were recapitulated: dominant 21-/22-nt length and 5′-uridine bias, consistent with DCL1 dependent biogenesis and AGO loading preferences (Mi et al., 2008; Montgomery et al., 2008).

While many conserved miRNA families are shared across angiosperms, palm lineage specific novel miRNAs may contribute to the unique stress biology of tropical palms. Comparative miRNA profiling in the closely related coconut (*Cocos nucifera*) under cold stress has documented a mixture of conserved and coconut specific stress responsive miRNAs (Li et al., 2024), highlighting that Arecaceae palms deploy both deeply conserved and lineage-restricted miRNA regulators when encountering cold stress.

### 4.4 Cold stress-related miRNAs in oil palm

Using Upset intersection analysis combined with Mfuzz fuzzy c-means time series clustering (Kumar and Futschik, 2007), we resolved two complementary layers of miRNA cold response: a core cold responsive miRNA set modulated across all five cold comparisons, and multiple stage specific miRNA sets only differentially expressed at discrete time points, further partitioned into six unbiased temporal expression modules (M1-M6). The early transient module M1 peaks specifically at 0.5 h, matching the physiological early perception window before major membrane damage; other modules capture mid-phase repression, sustained up-regulation and sustained down regulation trajectories.

Several conserved miRNA families we detected are well documented in abiotic stress responses across monocots. Eg-miR393 was progressively induced during cold exposure in oil palm. miR393 modulates auxin signaling via targeting TIR1/AFB auxin receptors, yet its cold response behavior is species dependent: it is cold induced in *Arabidopsis* and switchgrass but repressed in wheat under cold conditions (Gupta et al., 2014; Sunkar et al., 2007). Eg-miR166 family members were consistently down regulated under cold stress, correlating with elevated transcript abundance of HD-ZIP transcription factor targets *ATHB9-like*/*ATHB12-like*. miR166-HD-ZIP regulatory modules participate in cold and drought responses across multiple monocot species including blueberry, wheat and coconut (Kantar et al., 2011; Li et al., 2022; Li et al., 2024). Eg-miR156 was also cold-induced in our dataset, consistent with observations in sugarcane under low temperature stress (Xin et al., 2010).

When compared with coconut cold response miRNA profiles (Li et al., 2024), we observe partial overlap of stress responsive conserved miRNA families (miR156, miR166, miR393), indicating shared regulatory logic within Arecaceae palms. At the same time, a large number of novel oil palm specific cold responsive miRNAs (42 core novel miRNAs plus multiple stage specific novel members) were detected, pointing to lineage specific post transcriptional regulation that may underpin the distinct cold sensitivity of oil palm relative to more cold tolerant coconut varieties. It should also be noted that members within one miRNA family exhibited divergent expression trajectories, indicating sub-functionalization among miRNA isoforms during cold stress adaptation (Devi et al., 2013; Wang et al., 2014). While qPCR validated 17 representative miRNAs, the full repertoire defined by UpSet and Mfuzz clustering provides a genome wide view of temporal miRNA reprogramming.

### 4.5 Functional characterization of target genes of cold responsive miRNAs in oil palm

miRNA target gene functional enrichment pointed to multiple stress relevant pathways. Among these, the sesquiterpenoid and triterpenoid biosynthesis pathway showed significant enrichment across all five cold stress comparisons. We emphasize that this represents a testable working hypothesis rather than a fully proven conclusion, because the inference is built exclusively on in-silico miRNA target predictions for a small number of enzyme-coding genes (HMGCR, SQS, β-AS), without direct degradome evidence of transcript cleavage and without metabolome measurement within this present study.

As circumstantial independent support for this hypothesis, our groups’ previously published metabolome study on cold stressed oil palm documented alterations in terpenoid related metabolite pools under low temperature treatment (Wu et al., 2026). Terpenoid metabolites function in antioxidant defense, membrane modulation and hormone precursor synthesis in plants under abiotic stress (Liao et al., 2016; Vranová et al., 2013). Under cold conditions in other plant systems, low temperature can induce terpenoid biosynthetic gene expression and metabolite accumulation (Mareri et al., 2022). Therefore we hypothesize that miRNA mediated post transcriptional tuning of sesquiterpenoid and triterpenoid pathway genes may contribute to oil palm cold stress responses. Formal validation will require degradome sequencing and time-resolved metabolomics to confirm both miRNA target cleavage and corresponding metabolite changes.

Other enriched pathways point to layered stress responses: early time point enrichment for selenium compound metabolism hints at rapid antioxidant responses; plant-pathogen interaction pathway enrichment suggests crosstalk between abiotic cold signaling and biotic defense pathways (Zhu et al., 2023); late stage enrichment for mismatch repair pathways suggests cold associated DNA damage may accumulate with prolonged exposure. These target gene enrichments collectively suggest that miRNA guided post transcriptional regulation orchestrates multi-layer defense, metabolism and damage repair processes during cold challenge.

### 4.6 Enrichment of the sesquiterpenoid and triterpenoid biosynthesis pathway in oil palm under cold stress

KEGG enrichment analysis of miRNA target genes in oil palm under cold stress revealed that the sesquiterpenoid and triterpenoid biosynthesis pathway was significantly enriched (FDR-adjusted q< 0.05) in all cold stress comparisons. Rich Factor metrics demonstrated maximum enrichment magnitude after 8 h cold stress (**Supplementary Table S10**). Consistent with our above reasoning, this observation provides correlative support for our working hypothesis that terpenoid metabolism participates in oil palm cold stress responses, yet it cannot serve as definitive proof, given the reliance on computational target prediction alone.

Terpenoids are important secondary metabolites in plants. Previous studies have shown that they play multiple roles in plant stress responses, including antioxidant defense, signal transduction, and regulation of stomatal movement (Liao et al., 2016). Moreover, some terpenoids serve as biosynthetic precursors of phytohormones such as gibberellins, abscisic acid, and brassinosteroids, thereby helping to coordinate plant growth with stress responses (Vranová et al., 2013). Temperature is a key environmental factor affecting the accumulation of plant secondary metabolites. According to a review, when ambient temperature drops to 4-10 °C, various plants are induced to accumulate terpenoid secondary metabolites (Mareri et al., 2022). Cold stress promotes terpenoid synthesis through three mechanisms: modulating endogenous hormone levels (e.g., jasmonic acid and abscisic acid), affecting the expression of transcription factors (e.g., bHLH and MYB families), and regulating the expression of genes encoding key enzymes in the biosynthetic pathway (Mareri et al., 2022). In basil (*Ocimum basilicum*), cold stress significantly altered the expression patterns of genes involved in mono- and sesquiterpene biosynthesis, as well as the corresponding metabolite contents (Majroomi Senji and Abdollahi Mandoulakani, 2018).

From a metabolic perspective, the biosynthesis of sesquiterpenoids and triterpenoids primarily depends on the mevalonate (MVA) pathway. The rate limiting enzyme HMGCR (3-hydroxy-3-methylglutaryl-CoA reductase) and the key enzyme SQS (squalene synthase) play important roles in regulating metabolic flux in this pathway (Tholl, 2006). In the present study, the genes encoding HMGCR and SQS were predicted as targets of miR159/miR396 and miR172, respectively, suggesting that miRNAs may participate in the regulation of terpenoid metabolism by modulating the expression of these key enzyme genes. At the functional level, triterpenoids play important roles in plant defense and environmental adaptation (Augustin et al., 2011). Collectively, these findings support our testable hypothesis that miRNA dependent modulation of sesquiterpenoid and triterpenoid biosynthesis may be part of oil palm cold stress adaptation, and this requires further multi omics experimental validation.

### 4.7 An integrated working model linking miRNA temporal dynamics and physiological cold stress trajectories in oil palm

Combining physiological measurements, CIELAB color metric profiling, Upset intersection analysis, and Mfuzz time series clustering outputs, we propose an integrated working model describing miRNA mediated cold stress responses in oil palm. During the early perception phase (0.5 h) plants exhibit no macroscopic leaf injury, yet significant shifts in the total color difference index ΔEab already occur, indicating subtle biochemical perturbation before large scale membrane breakdown takes place. At the molecular level, the early transient M1 miRNA module reaches its expression peak alongside 0.5 h exclusive stage responsive miRNAs. Core cold responsive miRNAs are already differentially expressed at this time point, triggering rapid post transcriptional reprogramming that precedes measurable elevations in MDA content or electrolyte leakage; this phase mainly represents stress perception and the initiation of early adaptive signaling. Moving into the onset of injury phase (1–2 h), rising MDA concentrations and electrical conductivity mark the commencement of membrane peroxidation. The M2, M3 and M5 miRNA modules dominate transcriptional behavior, reflecting a mixture of up- and down-regulated molecular events, while 1 h- and 2 h-specific stage responsive miRNAs also become active. Conserved miRNAs including members of the miR156 and miR166 families display prominent expression shifts, putatively orchestrating stress signaling cascades by modulating their transcription factor encoding target genes. This time window functions as a physiological tipping point, in which adaptive stress responses co-emerge with the first detectable signatures of irreversible cellular injury. Finally, under the late severe damage phase (4–8 h), plants suffer extensive leaf browning and tissue necrosis accompanied by severe membrane lipid peroxidation, high electrolyte leakage, chlorophyll degradation and pronounced *ΔE*_ab_ values. The M4 (mid phase suppression with late rebound) and M6 (sustained down regulation) miRNA modules predominate, and a small subset of late-stage specific miRNAs are activated under these deteriorating physiological conditions. Importantly, miRNA expression changes observed from 4 h to 8 h cannot be attributed solely to dedicated cold acclimation programs, instead, they represent a composite signal in which residual adaptive transcriptional and post transcriptional reprogramming is superimposed on broad molecular perturbation triggered by oxidative damage and incipient cell death (Imran et al., 2026). Collectively, this working model demonstrates that miRNA dependent reprogramming is triggered extremely rapidly in the early perception phase, long before visible cold inflicted damage appears. As cold stress exposure continues, the miRNA regulatory landscape progressively integrates molecular signals originating from accumulating cellular injury. This framework establishes testable hypotheses to guide future functional characterization of the core and stage specific miRNA candidates identified in the present study.

### 4.8 Study limitations

It should be noted that all miRNA target gene pairs reported in this study are computational in silico predictions, and direct biochemical cleavage evidence from degradome sequencing or 5′ RLM-RACE is absent. Notably, the regulatory pairs of miR166 family members with predicted target genes *ATHB9-like* and *ATHB12-like* lack experimental validation of transcript cleavage. The negative expression correlation observed between miR166 and these two *ATHB-like* transcripts using qPCR only reflects transcript level abundance changes; such correlation cannot definitively confirm miRNA mediated mRNA cleavage, as mRNA levels can also be altered by independent transcriptional regulatory mechanisms. Further biochemical experimental work will be required in future studies to verify these predicted miRNAs target regulatory interactions in oil palm. The enrichment of sesquiterpenoid and triterpenoid biosynthesis pathway from predicted miRNA targets constitutes a working hypothesis, which requires degradome sequencing and metabolomic profiling for definitive experimental validation.

Several important experimental limitations should be acknowledged for the present work. First, all experiments were performed exclusively using seed derived Tenera (Dura × Pisifera F_1_ fruit form) oil palm seedlings, which exhibited cold sensitive phenotypes under our cold stress regime. Using only a single genotype cannot biologically separate true cold tolerance regulatory mechanisms from general chilling injury triggered molecular responses. A comparative framework employing contrasting cold tolerant/cold sensitive genotype pairs (such as the Tanzania × Ekona oil palm material cited in this manuscript) would enable more rigorous discrimination between injury related signals and adaptive tolerance related regulatory events, such comparative analysis is recommended for future follow up work. Second, although seedlings were sourced from one single seed lot to reduce genetic noise, seed propagated Tenera F_1_ hybrid seedlings carry residual genetic heterogeneity among siblings. Combined with destructive single time point sampling design, inter individual genetic variation among seedlings is confounded with cold treatment effects. Vegetatively propagated clonal oil palm material would remove this confounding genetic variance and would constitute an improved experimental system for future time series stress assays. Third, our experimental design lacks time matched ambient temperature control samples harvested at the same clock times as the cold stressed samples. All cold treated samples were collected between 08:30 and 16:00 local time, whereas the sole ambient temperature CK was collected only at 08:00. Consequently, we cannot fully disentangle cold stress driven molecular changes from circadian clock dependent transcript fluctuations over the 0.5–8 h time course. The rapid leaf browning observed within 4–8 h under cold conditions may be exacerbated by continuous illumination during cold exposure, as light can aggravate chilling associated oxidative injury in tropical plant species. Future time series cold stress experiments should include time of day matched ambient temperature controls to separate circadian effects from cold specific responses.

## 5 Conclusion

This study characterizes differentially expressed miRNAs and their target genes in oil palm under cold stress. Target genes of cold responsive miRNAs were significantly enriched in transcriptional regulation and secondary metabolic pathways, including sesquiterpenoid and triterpenoid biosynthesis. 17 cold responsive miRNAs validated via qPCR and grouped according to their expression patterns. Correlative expression analysis demonstrated that temporal shifts of these miRNAs align with physiological and biochemical alterations (membrane lipid peroxidation, chlorophyll degradation, morphological injury) occurring during cold exposure. While this work cannot fully resolve the complete molecular regulatory mechanism of oil palm cold tolerance, it identifies priority candidate miRNAs (e.g., *Eg-miR166*, *Eg*-*miR393*, *Eg*-*miR156*, *Eg*-*miR444*) for functional validation and molecular breeding of cold tolerant oil palm materials, supplying transcript level foundational resources for future mechanistic research on miRNA mediated cold stress response in tropical oil palm seedlings. Genome wide Upset intersection analysis and unbiased Mfuzz time-series clustering further defined the core cold responsive miRNA set and six temporal expression modules, providing a genome wide temporal framework for the cold stress response in oil palm.

## Data Availability

The datasets presented in this study can be found in online repositories. The names of the repository/repositories and accession number(s) can be found in the article/**Supplementary Material**.
